# A 36,200-year-old carving from Grotte des Gorges, Amange, Jura, France

**DOI:** 10.1038/s41598-023-39897-7

**Published:** 2023-08-09

**Authors:** Francesco d’Errico, Serge David, Hélène Coqueugniot, Christian Meister, Ewa Dutkiewicz, Romain Pigeaud, Luca Sitzia, Didier Cailhol, Mathieu Bosq, Christophe Griggo, Jehanne Affolter, Alain Queffelec, Luc Doyon

**Affiliations:** 1 CNRS, MCC, PACEA, UMR5199, Université de Bordeaux, 33615 Pessac, France; 2 Department of Archaeology, History, Cultural Studies and Religion, Centre for Early Sapiens Behaviour (SapienCE), University of Bergen, 5020 Bergen, Norway; 3 Centre Jurassien du Patrimoine, 39000 Lons-Le-Saunier, France; 4 École Pratique des Hautes Études-Paris Sciences and Lettres University, Chaire d’Anthropologie Biologique, 75014 Paris, France; 5 Geology and Paleontology Department, Natural History Museum of Geneva, 1211 Geneva, Switzerland; 6 Staatliche Museen zu Berlin, Museum für Vor- und Frühgeschichte, 10117 Berlin, Germany; 7 CReAAH, UMR6566, CNRS, Université de Rennes-1, 35042 Rennes CEDEX, France; 8 CRAL, UMR8566, CNRS, École de Hautes Études en Sciences Sociales, 75006 Paris, France; 9 Departamento de Antropología, Universidad de Tarapacá, 1010069 Arica, Chile; 10 Laboratorio de Análisis e Investigaciones Arqueométricas, Museo Arqueológico San Miguel de Azapa, 1010069 Arica, Chile; 11 CNRS, TRACES, UMR5608, Université Toulouse Jean-Jaurès, 31058 Toulouse CEDEX, France; 12 CNRS, EDYTEM, UMR5204, Université Grenoble Alpes, 73376 Le Bourget-du-Lac CEDEX, France; 13 Ar-Geo-Lab, 2000 Neuchâtel, Switzerland; 14 Artehis, UMR6998, Université de Bourgogne, 21000 Dijon, France; 15 Institute of Cultural Heritage, Shandong University, Qingdao, 266237 China

**Keywords:** Archaeology, Cultural evolution

## Abstract

The earliest European carvings, made of mammoth ivory, depict animals, humans, and anthropomorphs. They are found at Early Aurignacian sites of the Swabian Jura in Germany. Despite the wide geographical spread of the Aurignacian across Europe, these carvings have no contemporaneous counterparts. Here, we document a small, intriguing object, that sheds light on this uniqueness. Found at the Grotte des Gorges (Jura, France), in a layer sandwiched between Aurignacian contexts and dated to *c.* 36.2 ka, the object bears traces of anthropogenic modifications indicating intentional carving. Microtomographic, microscopic, three-dimensional roughness and residues analyses reveal the carving is a fragment of a large ammonite, which was modified to represent a caniformia head decorated with notches and probably transported for long time in a container stained with ochre. While achieving Swabian Jura-like miniaturization, the Grotte des Gorges specimen displays original features, indicating the craftsman emulated ivory carvings while introducing significant technical, thematic, and stylistic innovations. This finding suggests a low degree of cultural connectivity between Early Aurignacian hunter-gatherer groups in the production of their symbolic material culture. The pattern conforms to the existence of cultural boundaries limiting the transmission of symbolic practices while leaving space for the emergence of original regional expressions.

## Introduction

The discoveries made in the last two decades have made clear that artistic expressions reflecting symbolic behaviours have not emerged in Europe 40,000 years ago as a result of a cognitive revolution^1,2^, but appeared over a long time span in different regions of the world and expressed themselves in various and gradually more complex forms^3^. Early, isolated instances of abstract engravings are found at sites dating between 500 and 350 ka^4–6^ and they are more numerous and regionally circumscribed after 150 ka^7–19^. The creation of monumental structures in deep caves, possibly used for symbolic purposes, dates to 170 ka^20^. The use of the human body to convey symbolic meaning with personal ornaments is attested after 140 ka in North and South Africa^21^, as well as in the Levant^22,23^. Although mineral pigments are considered an ambiguous proxy for symbolic practices owing to their ethnographically attested use for functional purposes^24^, many authors consider that after their initial occurrence from 500 ka in Africa and 400 ka in Europe, their systematic use at Southern African sites after 160 ka supports their implications in symbolically mediated activities^24,25^, as demonstrated by the coating of personal ornaments with red ochre^21,26^, and its smearing on engravings^16^. The earliest possible symbolic markings on cave wall are found in Europe and date back to 63 ka^27^, while the oldest known figurative representations date back to 45 ka, as documented by several sites from the South-eastern Asia Pacific Islands^28–30^. In Europe, figurative paintings and engravings on blocks and cave walls are known since 39 ka, e.g.,^31–33^. The earliest three-dimensional carvings come from Swabian Jura sites, in Germany, and are attributed to the Early Aurignacian culture and dated between 40- and 38 ka^34,35^.

The earliest European carvings take the form of small figurines made of mammoth ivory representing animals, humans, and anthropomorphs. Their technological and stylistic homogeneity and sophistication support a well-established, regional, artistic tradition^35^ that doesn’t appear to have any contemporaneous counterparts, despite the Early Aurignacian being present across Europe, from Spain to Poland. The four known contemporary carvings from Europe strikingly differ thematically, stylistically, and technologically from the Swabian specimens. The so-called Trou Magritte “Venus” (Belgium)^36^ and the abri Blanchard “phallus” (France)^37^ are conical objects with a groove delimiting a rounded tip, with no equivalent in Germany. The Kostenki 14 “head” (Russia), also made of ivory, is a spherical object with a circular groove possibly representing a human neck^38^. The Galgenberg “Venus” (Austria) is made of green serpentine and depicts a human outline in two rather than three dimensions^39^. In contrast with the Aurignacian record, the following Gravettian technocomplex (32–26 ka), also present all over Europe, features three-dimensional carvings, representing mostly women, in a variety of styles, raw materials, and sizes^40^. This pattern raises the question of why a specific innovative artistic practice, such the production of three-dimensional carvings, may quickly become a well-established tradition in one region while not being adopted by neighbouring groups that are otherwise sharing the same cultural adaptation. Are we facing differential degrees of connectivity in distinct cultural domains among Early Upper Palaeolithic hunters-gatherers with strong similarities in hunting and domestic technologies, and less so in symbolic practices?

**Figure 1:**
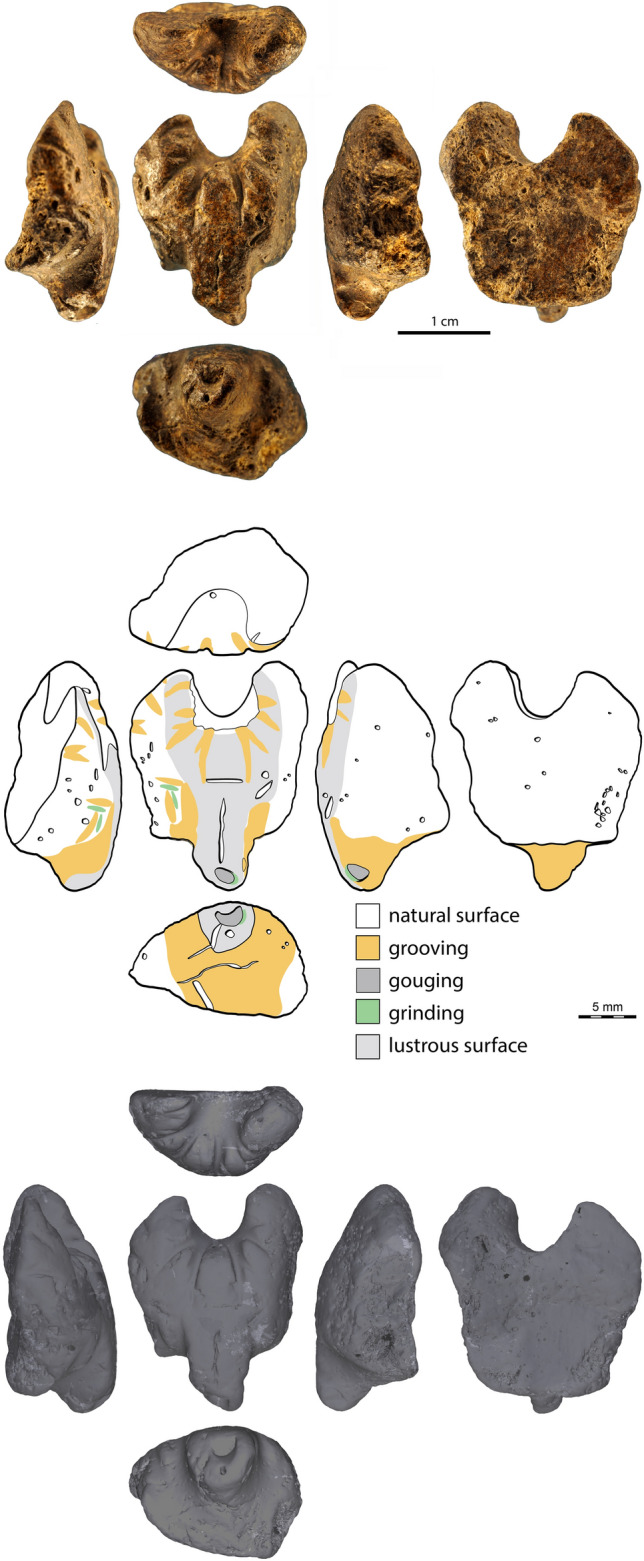
The Grotte des Gorges carving. Photographs (top), drawing (middle) and µCT surface rendering (bottom) of the Grotte des Gorges figurine.

Here, we document a small, intriguing object that sheds light on this issue. It was found at the Grotte des Gorges (Jura, France) in a layer dated to *c*. 36.2 ka, sandwiched between layers that yielded Aurignacian artefacts. The object bears traces of anthropogenic modifications suggesting intentional carving (Fig. 1). Microtomographic, microscopic and three-dimensional analyses reveal that this carving comes from a fragment of a large ammonite, which was modified to represent a caniformia head decorated with notches. While achieving a miniaturization comparable to that characteristic of the Swabian Jura carvings, the Grotte des Gorges specimen presents original features that suggest the craftsman emulated the ivory carvings whilst introducing substantial technical, thematic, and stylistic innovations. This behaviour supports a low degree of cultural connectivity between Early Aurignacian hunter-gatherer groups in the production of their symbolic material culture.

## Archaeological context

**Figure 2:**
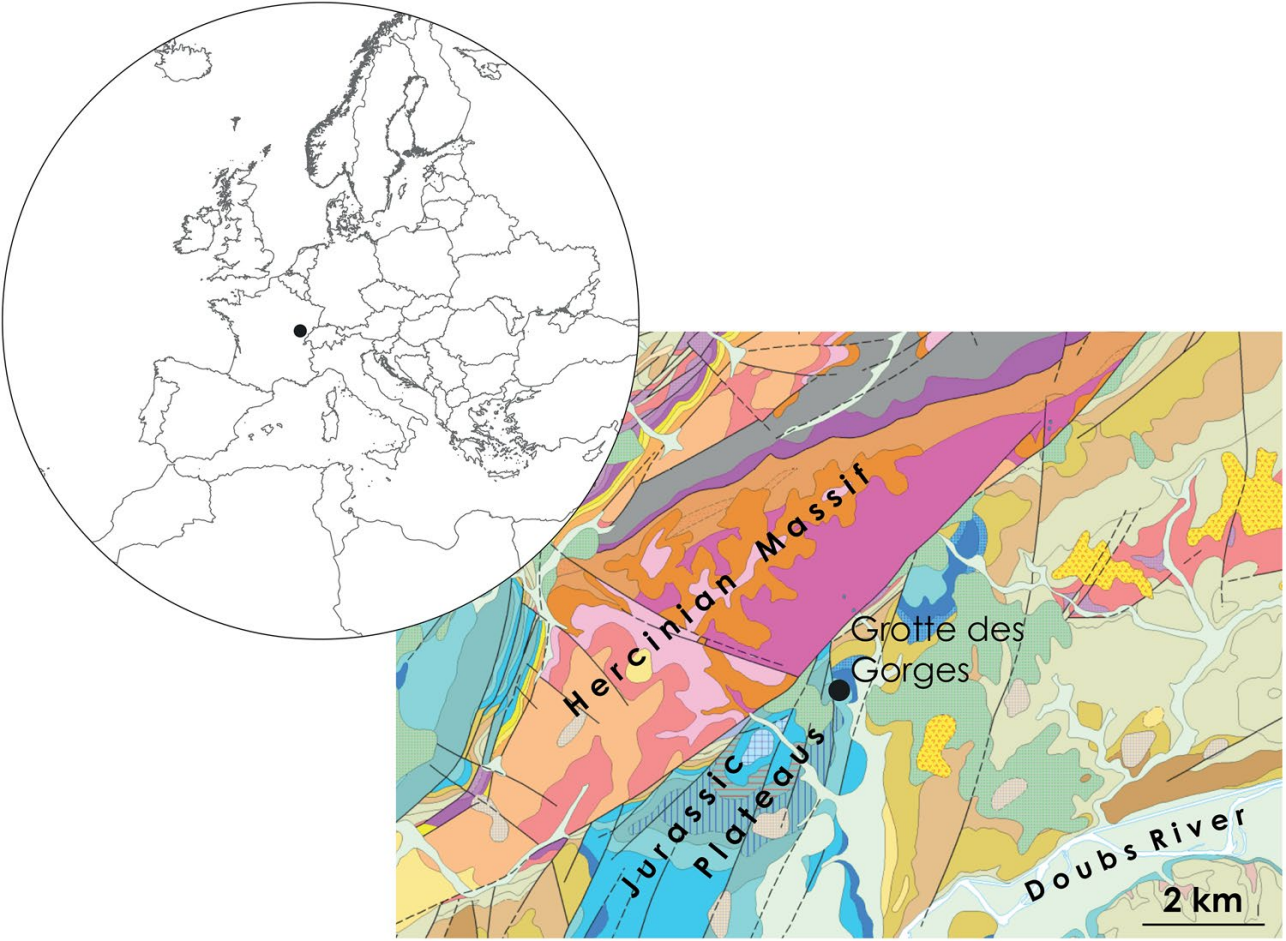
Site location and geological context of the Grotte des Gorges. Left: Geographic location of the Grotte des Gorges. Right: Vectorized geological map (1:50,000, Sheets ° 196, 1439). Created in QGIS 3.16.1 Hannover (www.qgis.org) from vectors and rasters available on Infoterre BRGM (www.infoterre.brgm.fr).

The Grotte des Gorges is a karstic cave developed on Jurassic formations located about 1 km southwest of Amange (Jura, France), along the southern slope of the Gorges valley. The valley is incised by the homonymous stream that originates in the Hercynian Serre massif (Fig. 2; SOM Text S1). The site was excavated from 2008 to 2017 under the direction of one of us (SD) over a surface of *c.* 35 m^2^ reaching a depth of 4 m. Sediments were water-sieved with a 2 mm mesh and all finds longer than 2 cm were piece plotted.

The most complete stratigraphic profile is observed in squares G10-12 and F12-13 excavated during the 2008–2009 seasons (SOM Figs. S1, S2; SOM Text S2). The five stratigraphic units (US) observed in that profile were grouped in a lower and upper sequence. The lower sequence includes US 4, 3, 2, and 1. US 4, 3, and 2 are interpreted as the result of mass movements of sediments accumulated outside the cavity and redistributed inside by debris flows and runoff processes. US 1, a rockfall unit, is only present at the entrance of the cavity. The upper sequence, consisting of US 0, is found only inside the cavity, and is composed of karstic clays originating from the surfaces of the slopes of the Serre massif. This deposit has almost filled the cavity (SOM Text S2).

US 4 yielded few faunal remains and eighteen lithic artifacts. Identified species comprise reindeer, bison, *Lepus* sp., woolly mammoth, red deer, and cave hyena (SOM Texts S3, S4; SOM Table S1). Lithics were scattered throughout the unit and consist of flakes, few retouched pieces, including a blade with typical Aurignacian retouch, a fragment of Dufour bladelet and a bladelet with direct marginal retouch (SOM Fig. S3; SOM Text S3). US 3 yielded a few worn bone fragments, a well-preserved horse rib, and the carving described in this study (SOM Text S3). US 2 encompasses three archaeological levels, from bottom to top, 2, 1b, and 1a. Levels 2 and 1b yielded remains of bison, reindeer, horse, steppe pila, root vole, and narrow-headed vole. A bone retoucher on a fragment of bison long bone diaphysis was found in level 1b (SOM Texts S3–S5) alongside few lithic micro-flakes (SOM Text S3). The presence of gnawed and regurgitated bone suggests that cave hyena played a role in the accumulation of the faunal assemblage from levels 2 and 1b (SOM Fig. S4; SOM Table S3). Anthropogenic traces in the form of cut marks and percussion notches indicate humans played a role in the accumulation of the assemblage as well (SOM Fig. S4; SOM Table S3). Level 1a records small, short-lived occupations. The fauna includes reindeer, bison, cave bear, a large undetermined ungulate, mammoth or rhinoceros, alpine ptarmigan, narrow-headed vole and three species of lemmings, reflecting a more rigorous environment than the previous levels (SOM Texts S4, S5). Systematic sieving allowed for the recovery of one hundred and twenty-eight lithics. Manufacturing by-products represent two-thirds of the assemblage. The remaining third comprises debitage products: flakes, blades, and bladelets. Nine bladelets or bladelet fragments were found. The three complete bladelets are straight and less than 2 cm in length. The assemblage also includes six blade fragments, including one with typical Aurignacian retouch, a bladelet core, and a massive flake retouched at its distal end that is interpreted as an Aurignacian nose scraper (SOM Fig. S5). An elongated, flat fragment of ivory bearing polish at one end was also found (SOM Fig. S5). US1 and US0 are archaeologically sterile (SOM Text S3). In US 4 and US 2, although good raw materials are locally available, a substantial proportion of lithics come from distant sources reaching up to 200 km. Flint transport indicates an origin from sources located 60 km to the south (Cesancey deposits), 207 km to the west (Yonne valley), and 165 km to the north-east (Liel-Schliengen, Germany) (SOM Text S6).

Engravings of Palaeolithic style depicting, among others, megaloceros, horse, proboscidean, and felines, were identified on the cave ceiling and on limestone blocs. Most engraved blocs were found on the surface. Three were uncovered at the interface between US 2 and US 0 (SOM Text S7).

A total of 20 bone remains from the Grotte des Gorges were radiocarbon dated (SOM Table S6). The sample from US 3 and another from US 2 level 2 were dated five and four times respectively. All ages are older than 32 ka aside from two. The first comes from a fragment of a human femur found outside the cave in square N6 within the upper portion of US 2. It yielded an age of 5,620 ± 30 BP (Beta-319487), which corresponds to the Neolithic period. The second was produced on a fragment of a bison humerus found inside the cave in square G13, US 2 level 1b. It yielded an age of 19,150 ± 170 BP (SacA-25148), which corresponds to the LGM.

The Bayesian model produced in Chronomodel 1.5.0^41^ identified the previous two ages as outliers and suggests calibrated age range (95% C.I., using IntCal20^42^) between 40,996 and 38,989 cal. BP for US 4, between 36,586 and 35,752 cal. BP for US 3, between 35,814 and 34,760 cal. BP for US 2 level 2, between 35,383 and 34,209 cal. BP for US 2 level 1b, and between 34,439 and 33,203 cal. BP for US 2 level 1a (SOM Figs. S10–S11).

The rare stratigraphic inversions observed in US 2 are likely the result of sedimentary dynamics and post-depositional disturbance by carnivores. Nonetheless, all ages fall within the age range known for the Aurignacian technocomplex in Western Europe^43^.

The object described in the present study was found in US 3 (SOM Text S8), which is dated between 36,586 and 35,752 cal. BP. Considering that the rib which has produced this age range was located 15 cm above the object, and that the minimum age of US 4 is 38,989 BP, the minimum age of the object should be comprised between 36.5 and 39.0 ka BP.

## Results

### Raw material and taxonomic identification

The µCT acquisitions reveal that, contrary to what one may have expected from its colour and appearance, the object is not made of osseous material (Fig. 1). The 3D reconstruction shows it is filled with thin, homogenous sediment containing randomly oriented tubular features (SOM Data S1). These features are identified as spicules of fossil sponges, some of which taking the form of 4-pointed stars^44,45^. This observation and consideration of its outer morphology indicate the object is a fragment of an ammonite inner mold consisting of a partially preserved septum of the phragmocone. The bilateral symmetry of the object follows the symmetry of the septum along the siphuncular tube (SOM Figs. S6 and S7).

When observed in transversal section, the fragment suggests an ammonite with a rather wide, sub-circular ventral area. Among the taxa present in the local Callovian formations^46,47^ (geological maps Pesme and Dole), some Pachyceratidae, e.g., *Erymnoceras*, bear similar characteristics.

### Description, technology, and wear

**Figure 3:**
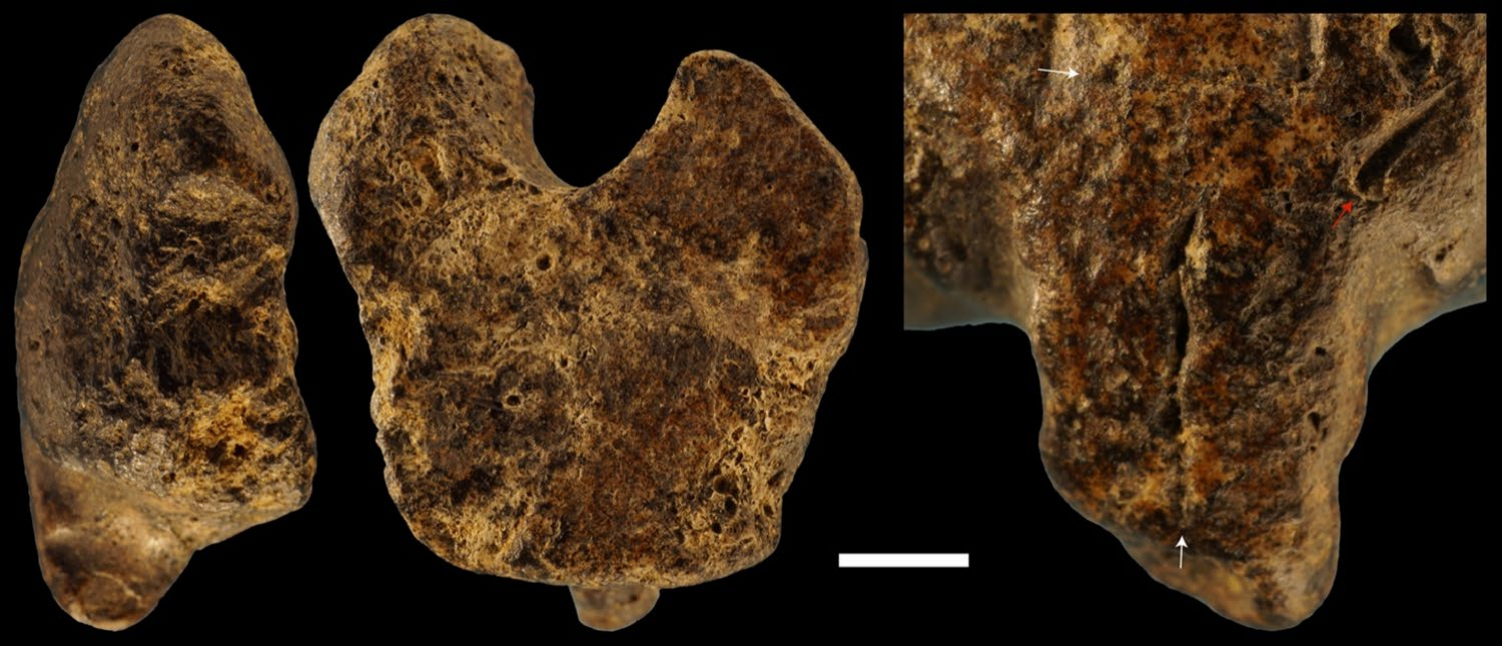
Natural surface of the figurine. The surface of the Grotte des Gorges figurine is covered with funnel-like openings due to the porous nature of the ammonite mould (left, centre). On the protuberance, two, non-joining, perpendicular, linear depressions (white arrows) with fringed outlines are interpreted as eroded imprints of the fossil. An oblique natural opening (red arrow) is located between the protuberance and the right edge (right).

In its present state of preservation, the object has an irregular plano-convex morphology with one side featuring a deep, round concavity and the opposite side presenting a protuberance (Fig. 1). The convex surface has a lustrous appearance. Microscopic analysis reveals the surface is covered with funnel-like openings due to the porous nature of the ammonite mould and present, on the protuberance, two, non-joining, perpendicular, linear depressions with fringed outlines interpreted as eroded imprints of the fossil (Fig. 3). Similar depressions are present on and below the protuberance.

**Figure 4:**
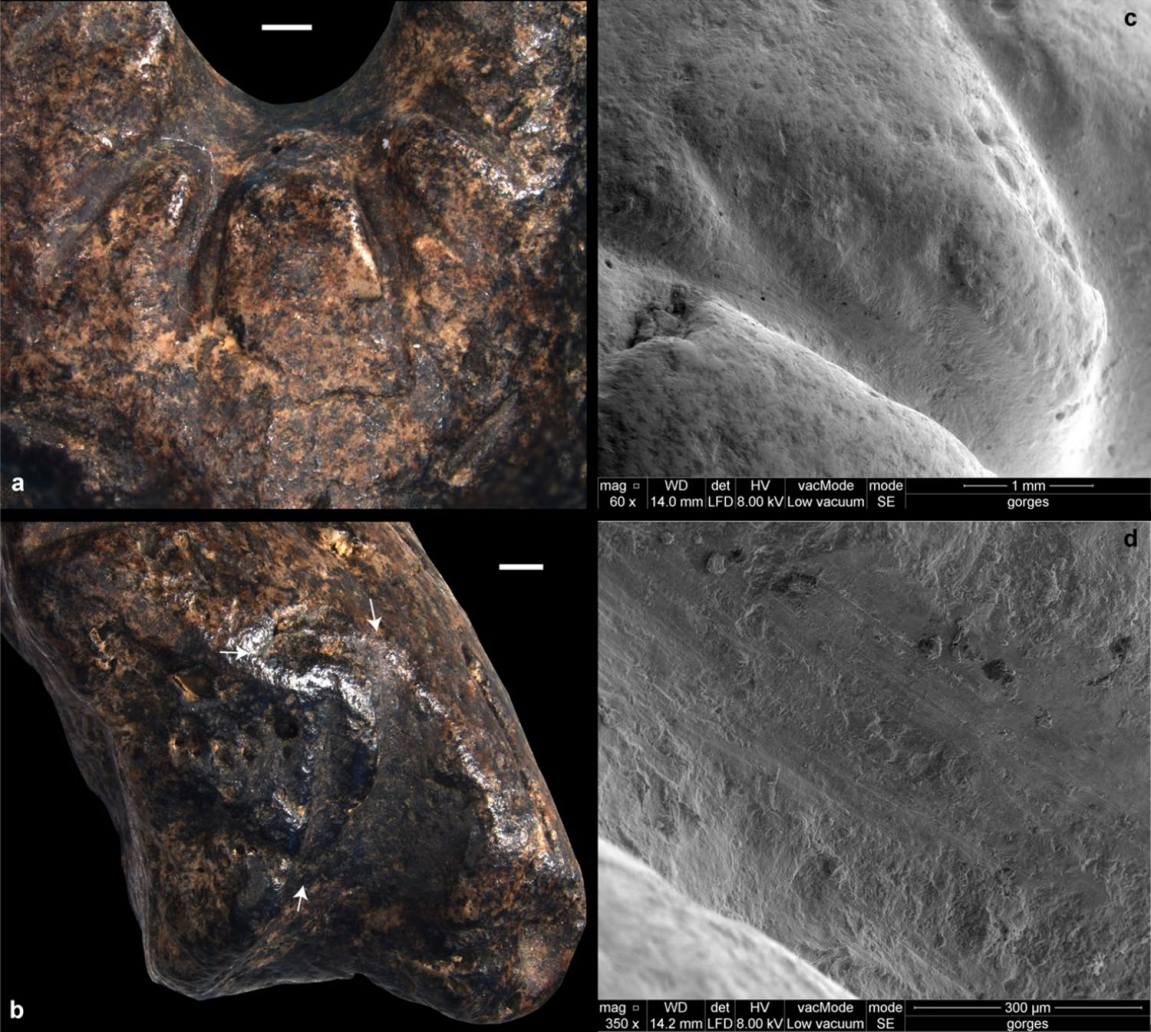
Traces of grooving. Close-up of the notches arranged radially around the concavity (**a**) and those present between the protuberance and the left edge of the figurine (white arrows) (**b**). These modifications result from the to-and-fro movement of an elongated edge. Parallel micro-striations are present inside the notches and suggest the use of a soft, quickly worn cutting edge, such as a limestone flake (**c**,**d**). Scales (**a**,**b**) = 1 mm.

**Figure 5:**
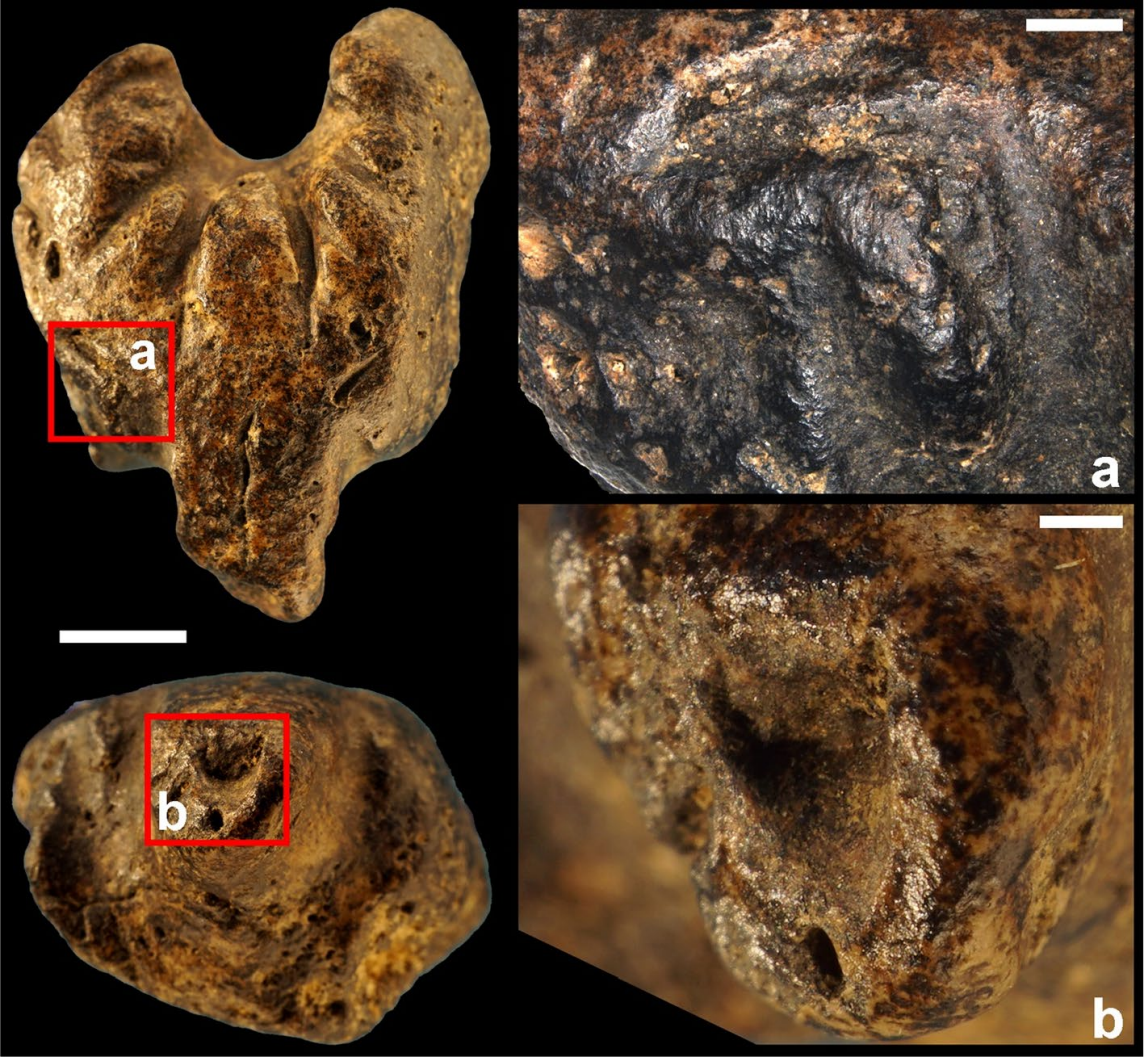
Traces of grinding and gouging. Grinding was applied to flatten protruding ridges between notches located on the left of the protuberance (**a**) as well as on the tip of the protuberance (**b**). Localized gouging applied on the tip of the protuberance created a concavity (**b**). Scales: general view (left) = 5 mm; (**a**,**b**) = 1 mm.

The ammonite fragment bears evidence of anthropogenic modifications by grooving, gouging, and grinding (Figs. 1, 4, 5). Grooving resulting from the to-and-fro movement of an elongated edge was applied around the concave area to produce 10 deep, sometime joining U-shaped notches arranged radially (Fig. 4). The same technique was used to incise four smaller notches on the left side of the object. Two small perpendicular notches were added midway between the protuberance and the left edge. The top one is positioned symmetrically to an oblique natural opening located between the protuberance and the right edge. On either side and below the protuberance, grooving was applied to make it more salient. All notches lack features produced when using a retouched or unretouched flint cutting edge in a to-and-fro motion, i.e., V- or irregular shaped sections with parallel micro-steps. The U-shaped section and superficial, sub-parallel micro-striations inside the notches (Fig. 4) rather suggest the use of a softer, quickly worn cutting edge, such as a limestone flake. The notches around the concavity are asymmetrical in section with those on the left being skewed toward the left and those on the right skewed toward the right (SOM Fig. S8). This is an indication that the object was turned 180˚ between the production of each set. A localized gouging was applied on the tip of the protuberance to create a concavity (Figs. 1, 5). The surrounding area was flattened by grinding. Grinding was also applied to flatten protruding ridges between the notches located on the left of the protuberance (Figs. 1, 5). Overall, the object bears 21 anthropogenic modifications: 17 notches of different types, three localized ground surfaces and one gouged pit.

**Figure 6:**
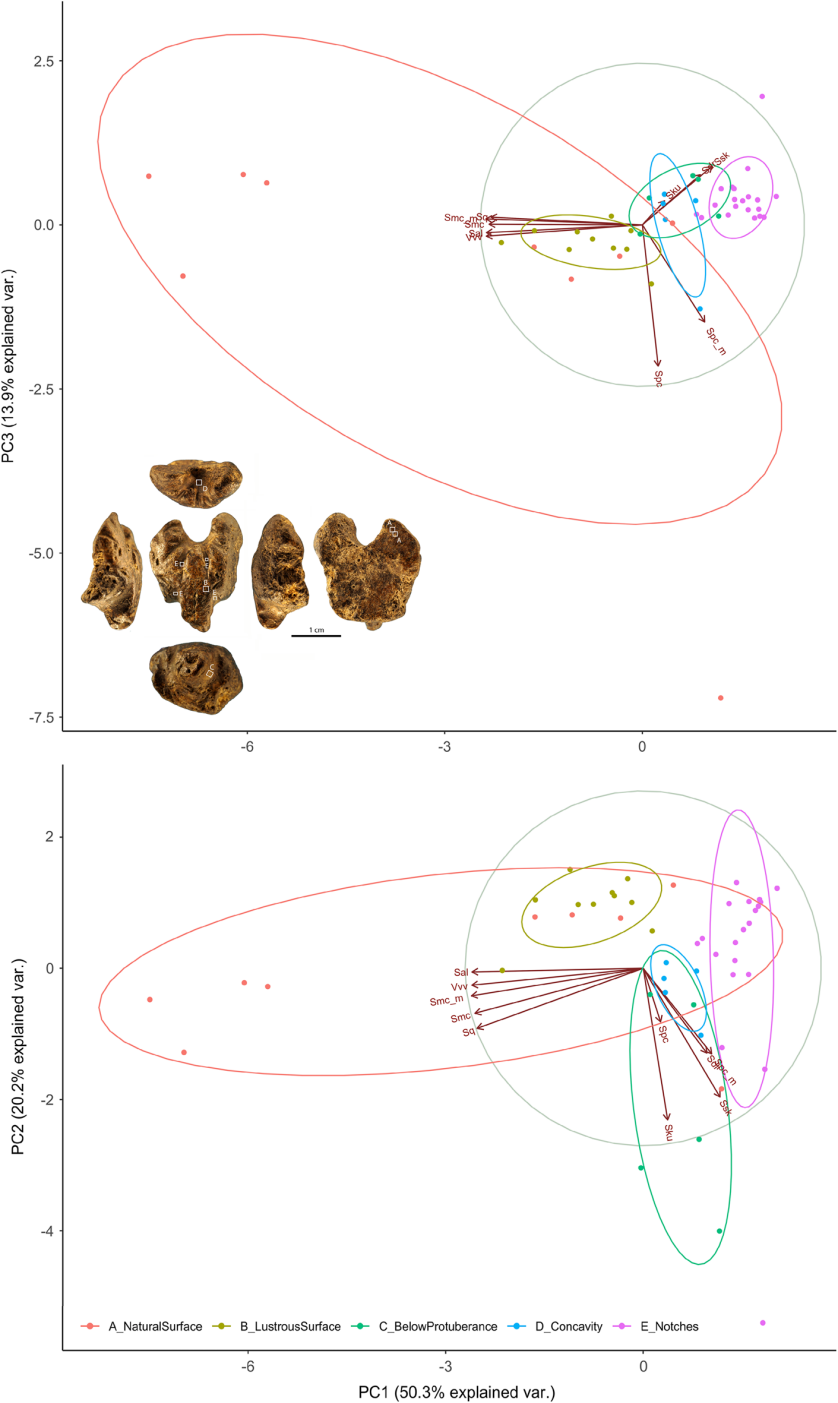
PCA of the textural data. The analysis demonstrates that the surface of the notches stands out in terms roughness while the highly polished area on the protuberance could represent an extreme in variation of the wear recorded for the remainder of the surface including the area below the protuberance interpreted as worked based on microscopic analysis. See Supplementary Tables S4, S5 for details.

Surface texture analysis identifies substantial differences between the surface of notches, the highly polished area on the convex face, the area below the protuberance, and the concave area on the opposite edge (Fig. 6; SOM Fig. S9; SOM Tables S4, S5). The natural surface roughness on the flat side of the object is highly variable and encompasses both the variation recorded on the broad concavity surrounded by deep notches and the lustrous area on the protuberance. Overall, the analysis demonstrates that the surface of the notches stands out in terms roughness while the highly polished area on the protuberance could represent an extreme in variation of the wear recorded for the remainder of the surface including the area below the protuberance interpreted as worked based on microscopic analysis.

**Figure 7:**
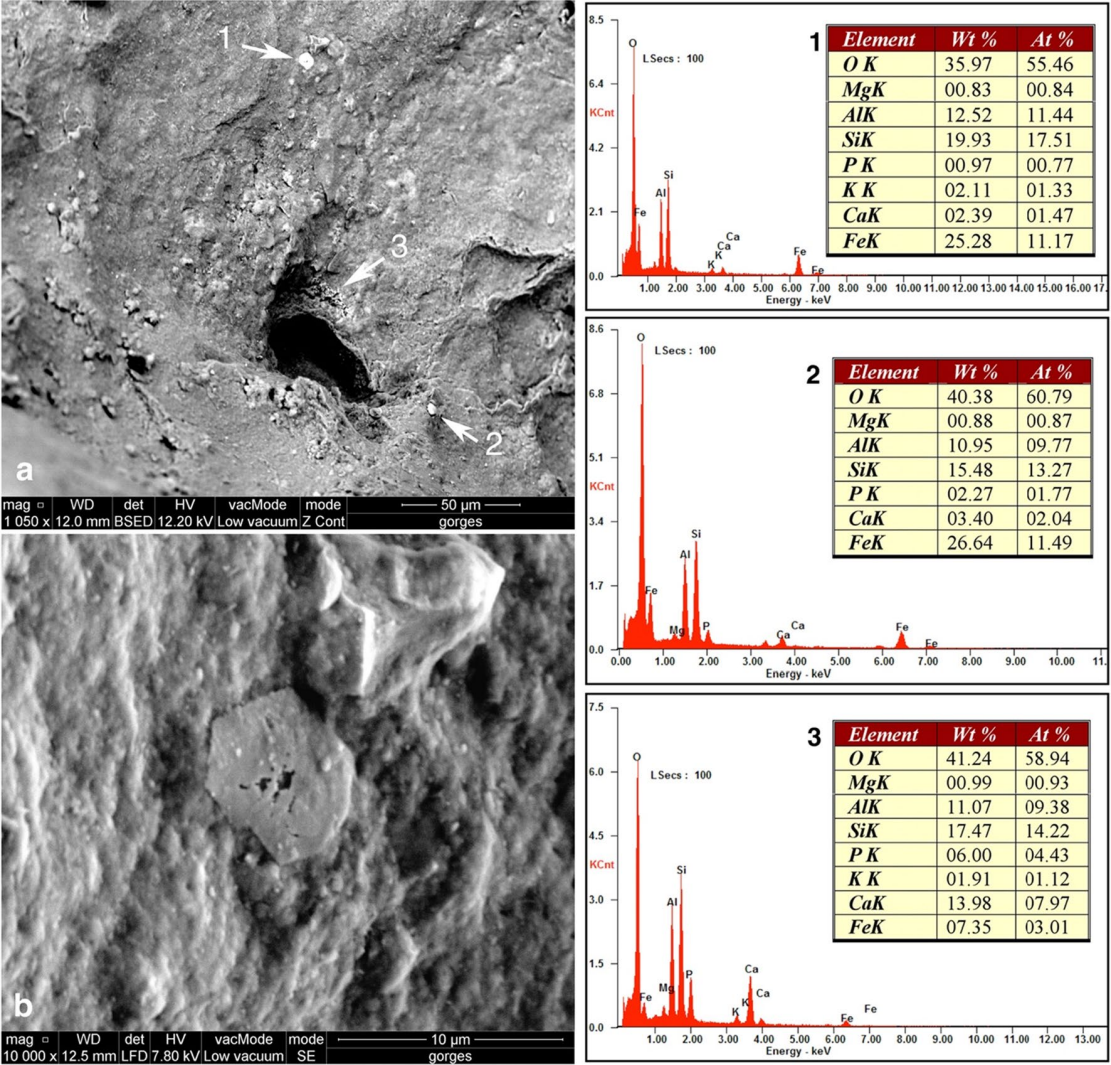
Chemical characterization of red particles present inside the notches arranged radially around the concavity of the Grotte des Gorges figurine. Acquisition location (**A**) and corresponding SEM spectra (right). Close-up view on the thin polygonal particle “a” (**B**). EDS reveals spectra “a” and “b” are richer in Fe_2_O_3_ than the spectrum “c” mainly composed of SiO_2_, CaO, Al_2_O_3_, and P_2_O_5_.

**Figure 8:**
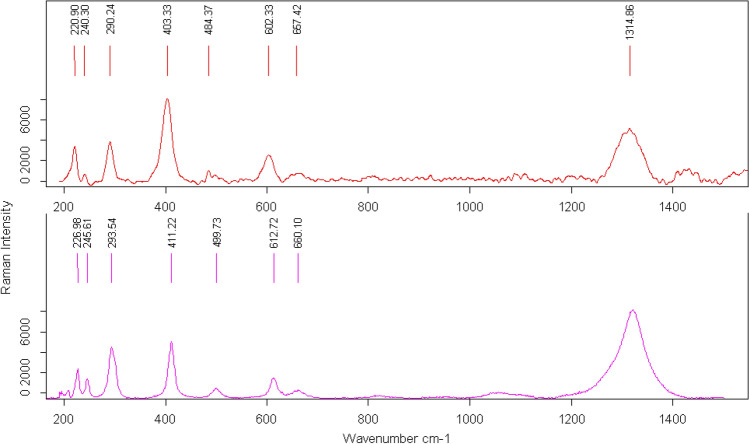
Raman spectra of the red particles present inside the notches arranged radially around the concavity of the Grotte des Gorges figurine. Representative Raman spectra of the red particles observed in the notches (top), compared with the reference spectra of hematite extracted from the RRUFF database (bottom). The position, width and relative intensity of the measured signal matches the reference spectra for hematite.

SEM analysis in BS mode of red residues detected at the bottom of the deep notches surrounding the concavity shows they consist of sub-circular particles ranging between 2 and 5 µm in size. EDS reveals they are richer in Fe_2_O_3_ than the surrounding surfaces composed of SiO_2_, CaO, Al_2_O_3_, and P_2_O_5_. A single thin polygonal crystal features a morphology compatible with hematite (Fig. 7). The presence of hematite particles is confirmed by Raman spectroscopy (Fig. 8). The US 3 and the sediment surrounding the object showed no detectable evidence of iron oxide. The presence of hematite particles inside the notches probably results from an ochre-rich compound originally highlighting the notches.

## Discussion

The modified ammonite fragment from Grotte des Gorges constitutes the first miniature carving interpreted as the representation of an animal head found in an Aurignacian site outside of the Swabian Jura. Its attribution to the Aurignacian is supported by the diagnostic lithics found below (US 4) and above (US 2) the stratigraphic unit (US 3) in which it was discovered. It is confirmed by the numerous broadly consistent radiocarbon ages dating the archaeological sequence (SOM Figs. S10, S11; SOM Table S6) and attributing an age of ~ 36,2 ka to the object. This attribution is also consistent with the faunal assemblages recovered throughout the stratigraphy (SOM Texts S4, S5) and the presence, in the layer caping the sequence, of slabs bearing engravings depicting Pleistocene fauna (SOM Text S7).

A symbolic use of the object is supported by its small size, unusual raw material, and the absence of traces indicative of a use as tool. The use of fossil ammonite for symbolic purposes is attested in the Aurignacian (SOM Text S9). The deep notches surrounding the natural concavity, arranged radially and symmetrically along the main axis of the object, originally filled with ochre, demonstrate the will to deliberately modify a natural shape while keeping its overall morphology. The natural shape was also modified by enhancing the pointed outline of the protuberance, carving a concavity at its tip, and regularizing it by grinding. Intriguingly, a groove was added to create a symmetry with a similar, albeit natural, linear depression present on the opposite side of the protuberance. These last modifications evoke the possibility that their production was aimed to represent the muzzle, snout, and eyes of an animal, and the natural protrusions on either side of the opposite concavity possibly representing ears.

Although clearly recognized by the microscopic analysis, the minimalistic nature of these modifications makes the identification of the depicted species speculative. The proportion and extension of the modified protuberance and the location of the possible eyes seem to rule out felids and rather evoke a member of the caniformia suborder such as bear, fox, wolf, or wolverine. The fact that the snout was slightly flattened by grinding perhaps makes bear the most likely represented species. Representations, including carvings, of bear (SOM Text S9) are known in Aurignacian contexts. The clear differences in surface texture between the highly polished appearance of the snout, the carved notches, and the remainder of the surface are consistent with an object transported over an extended period, the shiny appearance of the snout being the possible result of localized manipulation. The identification of hematite-rich ochre in the notches may originate from the object being transported in an ochred container or from being covered with ochre. The former hypothesis is the most likely considering the dark colour of the raw material, which absorbs the red colour rather than contrasts with it.

The identification of an Aurignacian miniature carving, possibly depicting a caniformia head, in a region 300 km away from the closest Aurignacian sites that have yielded abundant ivory carvings raises the question of why a such specific innovative artistic practice, i.e., the production of three-dimensional carvings, may quickly become a well-established tradition in one region while not being widely adopted by neighbouring groups otherwise sharing the same cultural adaptation in hunting and domestic technologies. The absence of suitable raw material does not appear to be a viable explanation since ivory was found at the Grotte des Gorges (SOM Text S3; SOM Fig. S5) and numerous Aurignacian sites in France and Belgium^48,49^. Therefore, the reason most likely lies in the structuring and connectivity of Aurignacian communities and the way in which cultural innovations spread between them.

Interestingly, the provenience of flint transformed into lithic tools found at the Grotte des Gorges shows no connection with the Swabian Jura. The Aurignacian hunters-gatherers who visited the site collected flint from sources, or acquired it through exchanges, over 60 km to the south, 165 km to the north-east, and 207 km to the west. This observation is consistent with variation in personal ornament types found at Aurignacian sites. Those found at the sites closest to Grotte des Gorges, e.g., Trou de la Mère Clochette and Grotte du Renne, display some similarities with those recovered in the Swabian Jura but also with those found in Belgium, the Rhone Valley and southwestern France^50^. One reaches the same conclusion when contrasting the origin of ochre used during the Aurignacian in the Swabian Jura. Recent analysis indicates that although ochre acquisition from distant sources is attested, there is a clear preference for local sources^51^. These patterns conform to the existence of cultural boundaries shaping the transmission of symbolic practices while not preventing cultural contact in the domains of technology and adaptation.

We argue that the production of the Grotte des Gorges carving better fits a case of emulation rather than imitation, sensu^52–54^. Emulation refers to the process of learning by focusing on the goals or outcomes of a behavior rather than imitating the specific actions or techniques used to achieve those goals. It involves identifying the desired results to achieve and pursue similar goals through their own means. Imitation involves replicating the specific actions, behaviors, or techniques of others. It focuses on reproducing the behavior itself, often through observational learning, and can be considered a more faithful reproduction of the model's actions. In the case of the Grotte des Gorges, emulation is suggested by the choice of a raw material that, contrary to mammoth ivory, has largely determined the final morphology of the figurine, the minor role played by shaping, and the original way in which deep radial grooves were applied to modify the object. Although the final goal is shared between the Grotte des Gorges and the Swabian Jura figurines, the means to achieve them differed drastically.

## Methodology

Taxonomic identification of the fossil used to make the carving was based on comparisons with reference collections curated at the Natural History Museum of Geneva, Geology and Paleontology Department, and criteria established in the literature^55–57^. The carving was photographed with a Sony ILCE-6500 equipped with 3.5/30 mm macro-lens and a motorized Leica Z6 APOA equipped with a DFC420 digital camera linked to LAS Montage and Leica Map DCM 3D computer software. Photographs of the carving were imported into Adobe Illustrator to make a tracing recording area bearing traces of manufacture and wear identified under microscope. The origin of these modifications was inferred based on criteria published in the literature on stone, bone, and ivory carvings^35,58–60^. The identification of these traces was cross validated by three of us (ED, FdE, LD).

Microtomography acquisitions were performed at the PLACAMAT laboratory (University of Bordeaux) using a Phoenix NANOTOM® GE with the following scanning parameters: 70 kV, isotropic pixel size of 18 µm (leading to a voxel size of 18 µm). Images were reconstructed with the TIVMI® v2.3 software based on the HMH (*Half-Maximum Height*) algorithm^61^ extended to 3D^62^. Segmentation features present in the infilling was performed manually. Although time consuming, it was facilitated by differences in density between the material constituting the carving and voids in the infilling. The acquired 3D data was used to create an interactive PDF allowing to view the external mesh, the internal features or both.

High-resolution surface topography of selected areas was obtained using a MarSurf CM mobile confocal microscope driven by MarSurf MSW 8.6 software. This equipment was used with the aim of producing 3D renderings and profiles of natural and anthropogenic traces and characterize the roughness of worn and unworn areas. Post-acquisition treatment was carried out using the Mountain View 8 software and followed a procedure adapted from Martisius et al.^63^, d’Errico et al.^64^ and Ma et al.^65^. Using built-in operators, it entails levelling the surface (least square method), removing outliers (both isolated and close to the edge), and filling-in non-measured points (interpolating values from neighbors). Profiles of anthropogenic traces were extracted at this stage. In the case of worn and unworn surfaces, the form of the 3D acquisition was also removed (polynomial of fifth order). The roughness parameters (ISO 25178) extracted from the acquisition include three height parameters, i.e., *Sq*, *Ssk*, *Sku*, one spatial parameter, i.e., *Sal*, one hybrid parameter, i.e., *Sdr*, one functional surface parameter, i.e., *Smc*, one functional volume parameter, i.e., *Vvv*, and one feature parameter, i.e., *Spc* (see SOM Table S4 for a detailed description of these parameters). Since tribology is concerned with interactive surfaces relative to motion, the ISO 25178 roughness parameters mainly provide information on the upper portion of the surface, i.e., on peaks, and no equivalent parameters to *Smc*, and *Spc* is available for the valleys. To circumvent this limitation, the worn and unworn surfaces were mirrored on their z-axis, and *Smc* and *Spc* were extracted a second time. To avoid confusion, the two parameters extracted on the mirrored acquisition were labeled *Smc_m* and *Spc_m* (SOM Fig. S9; SOM Tables S4, S5).

Areas with reddish residues were analyzed with a JEOL JSM-6460LV SEM. The observations and analyses were conducted under a low vacuum mode by using an accelerating voltage of 20 kV. Element analyses were carried out with an EDS INCA Oxford 300 spectrometer. During the analyses, the working distance was kept constant (8 mm). Acquisition time was set up to 60 s for each EDS spectrum with an average downtime of 40 per cent. Raman analysis of red particles was conducted with a SENTERRA Dispersive Raman Microscope. The analyses were done with a 785 nm laser and a laser power of 1 mW to avoid thermal photodecomposition on the analyzed area. Acquisition time was set to 20 s and multiple co-additions to improve the signal-to-noise ratio. The spectrometer worked in a spectral range from 65 to 2980 cm^−1^. The working areas were observed through an integrated color camera INFINITY1. Data were collected and processed with OPUS v. 7.2 (Bruker Optik GmbH, Ettlingen). The mineral phase identification was based on comparisons of the recorded spectra with those of the RRUFF database^66^.

### Supplementary Information

Supplementary Information 1.

Supplementary Information 2.

## Data Availability

All data are available in the main text or the supplementary materials.
