# Supplementary material for: A 36,200-year-old carving from Grotte des Gorges, Amange, Jura, France
Source: Sci Rep. 2023 Aug 9;13:12895. doi: 10.1038/s41598-023-39897-7 (PMC10412625; doi:10.1038/s41598-023-39897-7)
Supplement: Supplementary file 2 — Supplementary Information 2. [file 41598_2023_39897_MOESM2_ESM.pdf]

Supplementary Online Material for

A 36,200-year-old carving from Grotte des Gorges, Amange, Jura, France

Francesco d'Errico *et al.*

This Supplementary Online Material files contains:
Supplementary Text S1-S9
Supplementary Figure S1-S17
Supplementary Table S1-S6
References

Other Supplementary Online Material not included in this file:
Supplementary Data S1 (3D interactive PDF)

Table of content

Supplementary Text 2
Supplementary Text S1. Geological context 2
Supplementary Text S2. Stratigraphy and site formation processes 2
Supplementary Text S3. Archaeostratigraphy 4
Supplementary Text S4. Macrofauna and avifauna 5
Supplementary Text S5. Small vertebrates 5
Supplementary Text S6. Lithic raw material provenience 6
Supplementary Text S7. Palaeolithic engravings 6
Supplementary Text S8. Stratigraphic origin of the carving 7
Supplementary Text S9. Ammonites and bear representations in the Aurignacian 7
Supplementary Figures 8
Supplementary Tables 25
Supplementary References 31

Supplementary Text

Supplementary Text S1. Geological context

The cave is located about 1 km southwest of Amange (Jura, France), along the southern slope of the Gorges valley, incised by the homonymous stream that originates in the Hercynian Serre massif. This crystalline horst, characterized by facies of granite, gneiss and eurite[1], is dissected by drains that run perpendicular to its main axis and that cross the Jurassic terrain that lies on its southern edge. These drains cross the Jurassic plateaus along a NW-SE axis before joining the tributaries of the Doubs. The latter is the main river axis running along the southern flank of the Serre massif. The valley of the Gorges has a longitudinal profile characterized by a high and a low part, separated by a small projection of the limestone substratum plateau (Fig. 2). This morphology is related to the karstification of the Jurassic formations which exerts a control on the flows coming from the Hercynian grounds. The Gorges stream flows underground and its current resurgence is located in lower part of the valley, where it is cut on limestone with Polypiers and oolithic attributed to the Upper Oxfordian[1]. On the western slope, the bottom of the valley is separated from the plateau by an escarpment about 1 meter high at the head of the valley and 5 meters high in its middle. At present, the steep profile of this escarpment is largely masked by colluvium. Old karstic corridors have been cut by the digging of the valley, and the Grotte of Gorges corresponds to one of these corridors. The cavity is less than 14 meters in length and on average 6 m in width (SOM Fig. S1). The in-filling of the cave makes it difficult to evaluate its southern and western extensions. In 2013, partial removal of the in-filling at the bottom of the terminal room revealed small, narrow galleries sealed with clay. The main gallery shows erosion marks, i.e., channels and "gouge marks", indicating it is a karstic drain set up under a drowned regime with a flow direction from the entrance to the rear. At the end of the cavity, a flat ceiling resting on a stratification joint results from the readjustment of the karst during a wet episode.

Supplementary Text S2. Stratigraphy and site formation processes

The most complete stratigraphic profile is observed in squares G10-12 and F12-13 (SOM Fig. S2) excavated during the 2008-2009 seasons. It includes five main stratigraphic units named, from top to bottom, US 0 to US 4, grouped into an upper (US 0 and US 1) and a lower sequence (US 2, 3, and 4).

The lower sequence, comprising US4, 3, and 2, develops over a thickness ranging from 2.5 to 3 m. It corresponds to a massive, matrix-supported diamicton, characterized by a coarse fraction of limestone gelifracts with an average size ranging from 2 to 10 cm (SOM Fig. S12). The matrix is mainly composed of coarse sands with infracentimetric quartz and feldspars, interspersed with clayey fine sands. Under thin section, the mineralogical assemblage is mainly composed of angular limestone clasts, quartz, micas (biotite and muscovite), and feldspars. The clasts exhibit great variability in coarse fraction, ranging from biomicritic wackestone to oolithic and peloidal grainstone limestone. In the lower part of this sequence, several sandy lenses are interstratified within the diamicton (SOM Fig. S12). The main lens is characterized by a succession of elementary units with crossbedding, alternating with massive to current-rippled medium to coarse sand beds and thin, weakly organic sand-clay to silt-clay beds, ranging from 1 to 15 cm in thickness (SOM Fig. S12). Alignments of limestone gelifracts laid flat on some of these beds are visible. According to fabric measurements on gelifracts and their projection on the

Woodcock diagram[2], the clast fabric is planar (SOM Fig. S13). Based on the L vector[3] and the Rayleigh test, the clast orientation cannot be statistically considered unimodal, although a NE-SW oriented mode is more pronounced than other directions (SOM Fig. S13). Outside the cavity, the unit is characterized by a diamicton with metric limestone blocks and slabs overlain by reworked diamict deposits. Two test pits excavated outside the cave have identified the presence of blocks and slabs several meters north of the cave's current entrance. Inside the cave, a thick weathering crust, typically ranging from 5 to 70 cm in thickness, develops on the top of the lower sequence (US 2) except in the section on squares E15 to G15, where it is barely visible (SOM Fig. S14). This horizon has a very irregular lower contact and is characterized by a color ranging from reddish black to blackish red. It exhibits a variable cementation of the diamicton. The carbonate gelifracts present a crust indicating impregnation by phosphates associated with phosphate nodules in the plasma. Thin sections of this horizon locally show the complete disappearance of limestone clasts, with the contours of the voids being surrounded by laminated phosphate deposits. Coatings and impregnations by ferro-manganese products are also common. The microstructure is mostly vesicular, which may imply bioturbation.

The abundance of blocks and slabs observed outside the cave show that the entrance originally extended several meters south of the current dripline, while the topographic profile of the basement attest a local slope towards the rear of the cave. This configuration, as well as probably the presence of a double slope at the base of the ancient dripline, likely contributed to the redistribution of materials into the cavity. The gelifracts originate from the rockfall of the ancient shelter vault and the walls of the rocky cliff that flanks the cave on both sides, while the sandy fraction likely originated from the plateau which is locally covered by mixed detrital formations (eolian, fluvial, weathering of the Jurassic substrate) (SOM Fig. S15). Based on stratigraphic observations, the lower sequence is characterized as a diamictic facies and debris flows, or runoff are retained as the main sedimentary processes that contributed to the final accumulation of the sediments (SOM Fig. S16). Diamictic facies are commonly observed in slope deposits and may result from various sedimentary processes such as rockfall, solifluction, debri flows, or runoff[4]. Microscopic analysis of thin sections from matrix-supported diamicton of the lower formation did not reveal any features related to frost action. Also, the fabric characteristics of the deposits (SOM Fig. S13) do not fall into the variability expected for solifluction mass movements[5,6]. Thus, solifluction is excluded as a sedimentary process. Finally, the channelized sand intercalations with cross-stratification and current ripples are interpreted as runoff facies that indicate a hyper-concentrated flow regime[7].

The XRD analysis of the weathering crust surrounding a limestone block confirms the presence of phosphates represented by minerals of hydroxyapatite (apatite-(CaOH)). Quartz is also well represented on the spectrum (SOM Fig. S17). The precipitation of apatite is commonly observed in karstic contexts[e.g., 8,9]. The neogenesis of phosphate may result from the percolation of acid phosphate solutions derived from the decomposition of bat guano[10]. These solutions are responsible for the decarbonation and weathering of the surface of the gelifracts observed in the weathering horizon, which ranges from 5 to 70 cm thick at the top of the lower sequence.

The upper sequence, consisting of US 0 and US 1, is essentially composed of clayey to silty deposits (US 0) and a localized layer of collapse limestone slabs near the entrance of the cave (US 1). Its thickness varies but originally filled the cave almost up to the ceiling. From bottom to top, two main sub-units are distinguished: yellowish-brown silt clays that overlie the weathering horizon at the top of the lower sequence, and brown to dark brown clays. At the rear of the cave, the dark brown clays are characterized by a high abundance of organic matter,

animal, and plant, and by significant bioturbation, as evidenced by the lumpy sediment structure. Metric limestone slabs are also present within the upper sequence. Based on their arrangement and orientation (SOM Fig. S2), their collapse occurred after the deposition of the lower sequence but before the accumulation of the upper sequence. The upper sequence likely results of a runoff accumulation from the endokarst as well as from the cave entrance by low-flow regime resumption (diffuse runoff) of sediments from the plateau. In the latter case, a comparable functioning can be observed currently in cavities near the Grotte des Gorges where inputs from the plateau are mainly concentrated at the level of faults and/or fractures in the limestone substrate (SOM Fig. S15). The presence of marked bioturbation in the upper sub-unit attests to significant biological activity. The absence of cryoclasts and, in general, coarse fraction suggests stabilization of the environment during the formation of this sequence associated with milder conditions.

Supplementary Text S3. Archaeostratigraphy

US4. A small lithic assemblage and few faunal remains were found in this unit. Identified specimens include, in decreasing occurrences, reindeer (*Rangifer tarandus*), bison (*Bison priscus*), hare (*Lepus* sp.), woolly mammoth (*Mammuthus primigenius*), red deer (*Cervus elaphus*), and cave hyena (*Crocuta spelaea*) (SOM Table S1). Eighteen lithic artifacts were discovered, scattered throughout the unit. They include flakes and few retouched pieces, mostly blades and bladelets. Among the later, a blade with typical Aurignacian retouch, a Dufour bladelet fragment and a bladelet with direct marginal retouch on the left edge were found (SOM Fig. S3). Nearly one-third of the lithics show signs of mechanical abrasion.

US3. This stratigraphic unit yielded a few worn bone fragments, a well-preserved horse rib attributed to *Equus ferus* cf. *gallicus*, and the carving described in this study (SOM Table S1).

US2. Three archaeological levels were identified in US 2, from bottom to top, 2, 1b, and 1a. Levels 2 and 1b yielded almost exclusively animal remains. In decreasing occurrences, the identified specimens include bison (*Bison priscus*), reindeer (*Rangifer tarandus*), and horse (*Equus ferus* cf. *gallicus*). Bison also dominates the faunal assemblage from level 1b, and reindeer is slightly more abundant compared to level 2. A bone retoucher on a fragment of bison long bone diaphysis was found in level 1b alongside few lithic micro-flakes. The presence of gnawed and regurgitated bone suggests that cave hyena played a role in the accumulation of the faunal assemblage from levels 2 and 1b (SOM Fig. S4). However, anthropogenic traces related to butchery activities (cut marks, percussion notches) and lithic tool retouching indicate that the site was occasionally visited by humans (SOM Fig. S4). Located at the top of US 2, level 1a record small, short-lived occupations. The fragmented fauna includes reindeer, bison, cave bear, a large undetermined ungulate, mammoth or rhinoceros, and alpine ptarmigan. Systematic sieving allowed for the recovery of one hundred and twenty-eight lithics. Manufacturing by-products represent two-thirds of the assemblage. The remaining third comprises debitage products: flakes, blades, and bladelets. Nine bladelets or bladelet fragments were found. The three complete bladelets are straight and less than 2 cm in length. Six blade fragments, including one with typical Aurignacian retouch, a bladelet core, and a massive flake retouched at its distal end that is interpreted as an Aurignacian nose scraper. (SOM Fig. S5) An elongated, flat fragment of ivory bearing polish at one end was also found (SOM Fig. S5).

US1 and *US0.* These two units are sterile.

Supplementary Text S4. Macrofauna and avifauna

A total of 667 remains were taxonomically and anatomically determined. The assemblage includes 21 taxa (SOM Table S1). Two species dominate the entire sequence: the steppe bison, *Bison priscus* (US 2 - levels 1b and 2) and the reindeer, *Rangifer tarandus* (US 2 - level 1a). Horse remains, *Equus ferus* cf. *gallicus*, although rarer, are present in almost all levels. Other herbivores, carnivores and birds are represented by a few remains (SOM Table S1).

Overall, the bones collected from the Gorges cave are relatively well-preserved. Their surfaces show no evidence of weathering or frost nor root etching.

The faunal assemblages from US 3, in which the carving was found, and US 4 are too small to propose a comprehensive interpretation concerning the agents responsible for their accumulation and subsequent taphonomic history.

The largest bone assemblage, from US 2, is characterized by a large proportion of isolated teeth and fragments of long bone diaphyses, particularly in levels 1b and 2 (SOM Table S2). Cranial fragments, vertebrae, ribs, long bone epiphyses, carpal and tarsal bones, and phalanges are rare. A difference is also observed in the sizes of bone fragments, which either fall into small-sized splinters averaging less than 2 cm in length or relatively thick and large diaphyseal fragments of Bison limb bones measuring *circa* 10 cm in length.

One-quarter of the diaphysis fragments are gnawed (SOM Fig. S4; SOM Table S3) either at one or both ends, by a relatively powerful carnivore in the size of a cave hyena. The presence of regurgitated bone fragments longer than 4.5 cm in length is consistent with this interpretation. The few hyena remains collected in levels 1a and 1b suggest that this carnivore also played a role in the bone accumulations of these levels.

The traces of anthropogenic origin are relatively rare (SOM Table S3). They are limited to a few butchery marks and percussion notches. However, a fragment of a bison long bone diaphysis is particularly interesting because the observed marks leave no doubt as to their anthropogenic origin. It has numerous defleshing marks, two percussion impacts, and an area covered with scars indicating that this piece was used as a retoucher to shape a lithic tool (SOM Fig. S4).

In Paleolithic sites corresponding to hyena dens, the proportions of remains attributed to this carnivore are always relatively high, and they are always associated with numerous coprolites and gnawed or regurgitated bones, as well as remains of hyenon[11–16]. While gnawed and regurgitated bones two have been identified in US2 at the Grotte des Gorges, only 8 faunal remains are identified as hyena (1.6% of the determined remains), and no coprolites were found in this unit. Hyena could therefore have frequented the site without having established a long-term den.

Few bone remains bear anthropogenic traces related to butchery activities or lithic tools shaping. The small lithic assemblage of US2 suggests the site or its entrance was intermittently used by human groups. Carnivores also visited the site and modified the bone remains left therein.

Supplementary Text S5. Small vertebrates

No remains of small vertebrates were found in US 3, in which the carving was discovered, nor in US 4.

The small vertebrate remains from US 2 are grouped into two assemblages. In the first assemblage, including levels 2 and 1b, 9 small mammal taxa have been identified including Eulipotyphla, such as mole or shrew, steppe pika (*Ochotona pusilla*), and voles, such root vole (*Alexandromys oeconomus*) or narrow-headed vole attributed to the extinct lineage *Lasiopodomys anglicus*[17]. The association of these taxa reflects wet steppe environments. Application to this faunal association of the Bioclim method[18,19] estimate mean annual temperatures of 4.5±6.5°C, and large thermal amplitudes of *circa* 25°C.

The second faunal assemblage from level 1a shows a similar association with the appearance of three additional lemming species, including the tundra lemming (*Lemmus lemmus*), the wood lemming (*Myopus schisticolor*)[20], and the collared lemming (*Dicrostonyx torquatus*). These rodents, currently living in the northernmost parts of Eurasia, are typical of tundra and boreal forests. Their presence is certainly related to a cold event such as a Greenland Stadial or an Heinrich event. This small mammal association suggests rigorous annual average temperatures, around -1.2±6.5°C, and important thermal amplitudes, in the order of 30°C.

Supplementary Text S6. Lithic raw material provenience

The provenience of the lithic raw material found at the Gorges cave was determined using the non-destructive method of identification of sedimentary microfacies (for details of the method, see[21,22]). Each specimen was observed of their non-prepared, wet surfaces under stereomicroscope at magnifications ranging from 10 to 80x. Other methods of determination based on geochemical or geophysical analyses (for example, see[23–25]) were neglected owing to the strong alteration and or encrustation on some surfaces.

The small lithic assemblage from US 4 includes seven variety of flint, and a flake of metaquartzite. A blade fragment is made of a flint probably originating from the north of the Parisian Basin. Five specimens come from the Upper Cretaceous of Cesancey (Jura, France), and one from the Oligocene of Mont-les-Etrelles (Haute-Saône, France). The remainder is local in origin: four from the Callovian, three from the Bajocian, and two from Triassic formations.

More than half (n = 66) of the lithic assemblage from US2, level 1a, present alterations preventing source attribution. Local flints dominate the assemblage and come from Bajocian and Callovian formations. Oligocene flint from Mont-les-Etrelles (Haute-Saône) is the most represented among the exogenous raw materials. Six elements come from the Malm reworked in the Eocene formation of Liel-Schliengen (Baden-Württemberg, Germany). One blade originates from the Cretaceous formation of the northern Parisian Basin.

In summary, although good raw materials are locally available, a substantial proportion of lithics come from distant sources reaching up to 200 km. Flint transport indicates an origin from sources located 60 km to the south (Cesancey deposits), 207 km to the west (Yonne valley), and 165 km to the north-east (Liel-Schliengen, Germany).

Supplementary Text S7. Palaeolithic engravings

Engravings of Palaeolithic style depicting, among others, megaloceros, horse, proboscidean, and felines, were identified on the cave ceiling and on 21 limestone blocs. On the ceiling, the engravings occupy a small space located in the western part of the cavity near square G17. Although most engraved blocs were found on the surface, three of them were uncovered at the interface between US 2 and US 0, near the western wall of the cave[26,27].

Supplementary Text S8. Stratigraphic origin of the carving

The carving described in this study was found by one of us (SD) in 2011. It comes from the middle of US3, in square E12. Its coordinates are x = 100, y = 45, z = -391 (SOM Figs. S1–S2).

Supplementary Text 9. Ammonites and bear representations in the Aurignacian

Ammonites were sometimes used as personal ornaments in the Aurignacian. Perforated specimens transformed in ornaments were found in five French sites: La Ferrassie, La Souquette and Les Festons in Dordogne, Les Rois in Charente and Rothschild rock shelter in Hérault[28,29]. One unperforated fragment of a lias ammonite from the Aurignacian layer V at Vogelherd bears seven notches on its edge[30].

A carving made of ivory representing a bear in an upright position comes from the Aurignacian level AH IIa at Geissenklösterle[30]. Another, missing its head and bearing five incisions on its right side, is documented in the Aurignacian material recovered by G. Riek at Vogelherd[31]. Several bears painted or engraved on cave walls, and attributed to the Aurignacian are found at the Micolón Cave[32] and Altxerri B Cave[33] in Spain, and Chauvet Cave[31,34] and the Grande Grotte d'Arcy-sur-Cure[31,35] in France. An engraving on limestone blocks depicting a bear was also found at the Blanchard rock shelter in Dordogne[31].

Supplementary Figures

Supplementary Figure S1. Plan of the excavated area at Grotte des Gorges. The location where the figurine was found is indicated with a red dot.

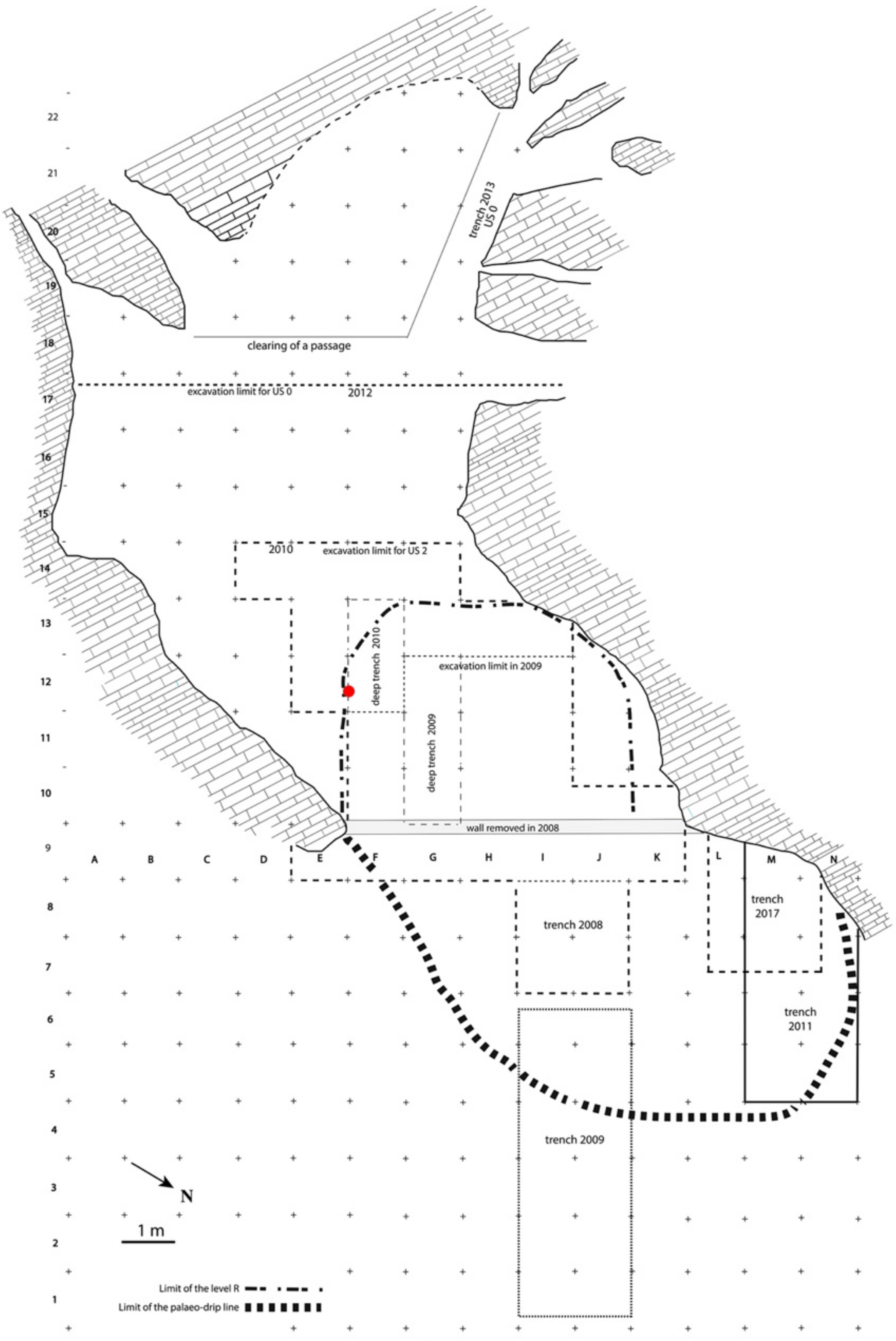

Supplementary Figure S2. Stratigraphy of the Grotte des Gorges with the location where the figurine was found (red dot).

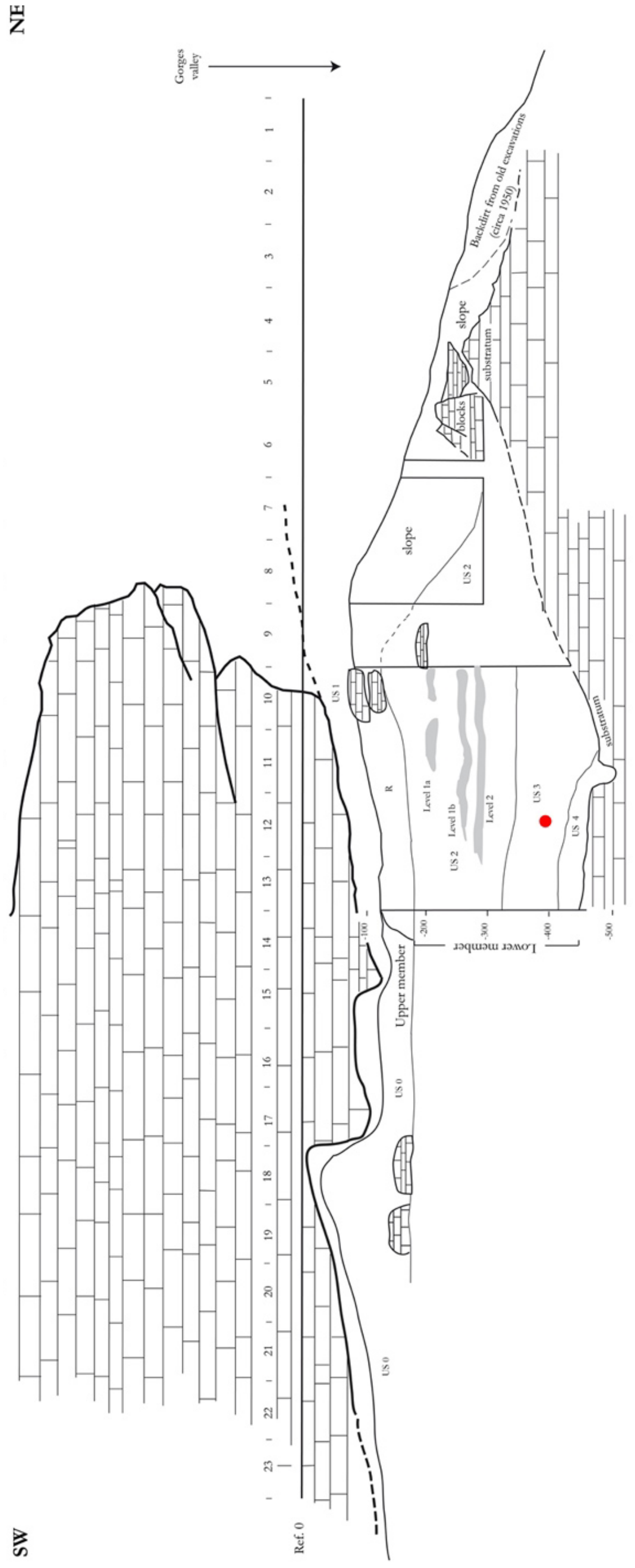

Supplementary Figure S3. Lithic tools from Grotte des Gorges US 4. Lithics were scattered throughout the unit and consist of flakes, few retouched pieces, including a blade with typical Aurignacian retouch, a fragment of Dufour bladelet and a bladelet with direct marginal retouch. Scale bar = 1 cm

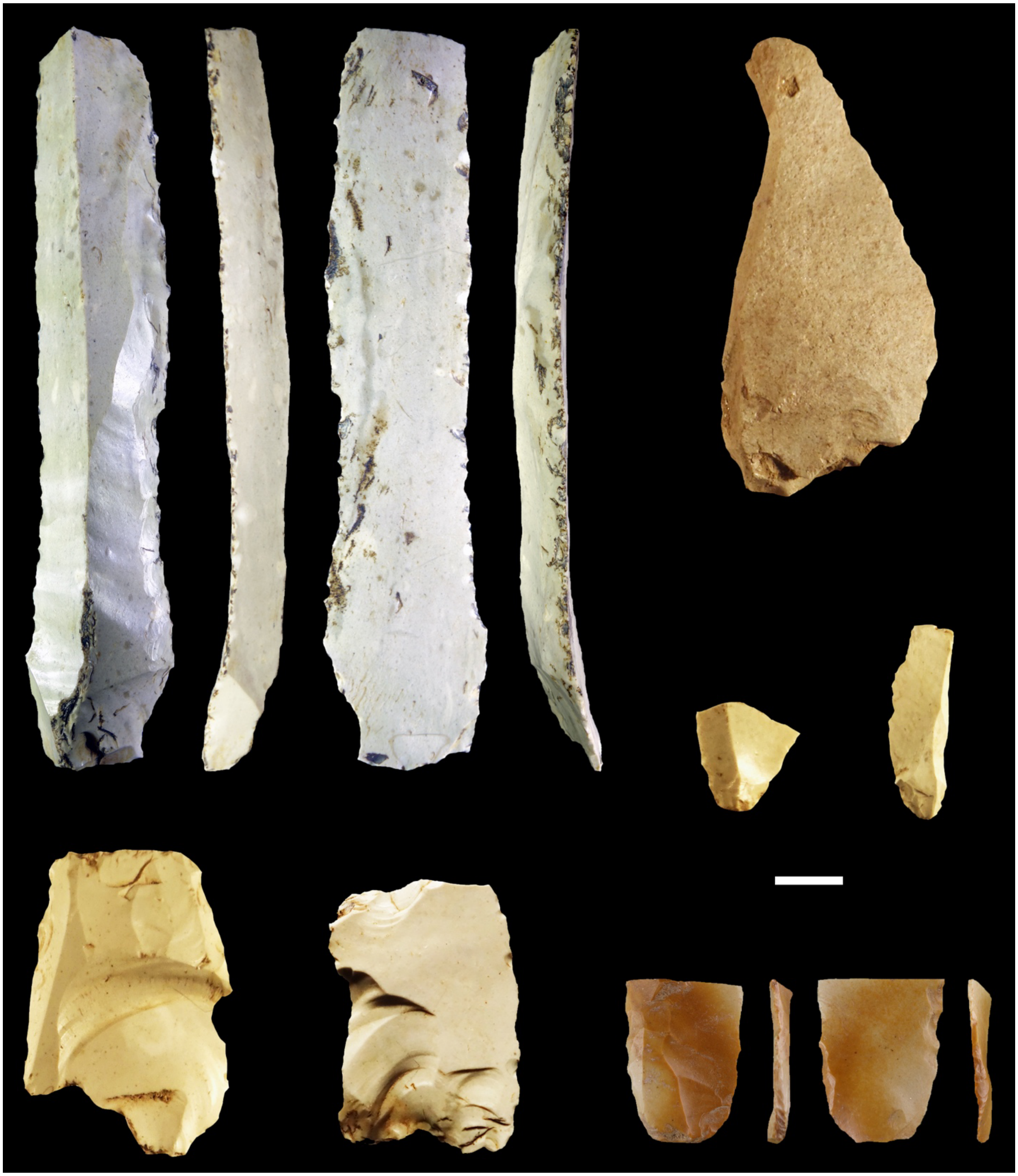

Supplementary Figure S4. Carnivore and human modifications observed on the faunal remains from Grotte des Gorges US 2. Bison bones bearing traces of gnawing (a) and evidence of regurgitation (a). A bison long bone shaft fragment found in US 2, level 1b (H10 n˚ 2) bears evidence of anthropogenic modifications (c) in the form of percussion notches (left), cut marks (right, top), and a pitted area attesting to its use as retoucher to sharpen lithic tools (right, bottom).

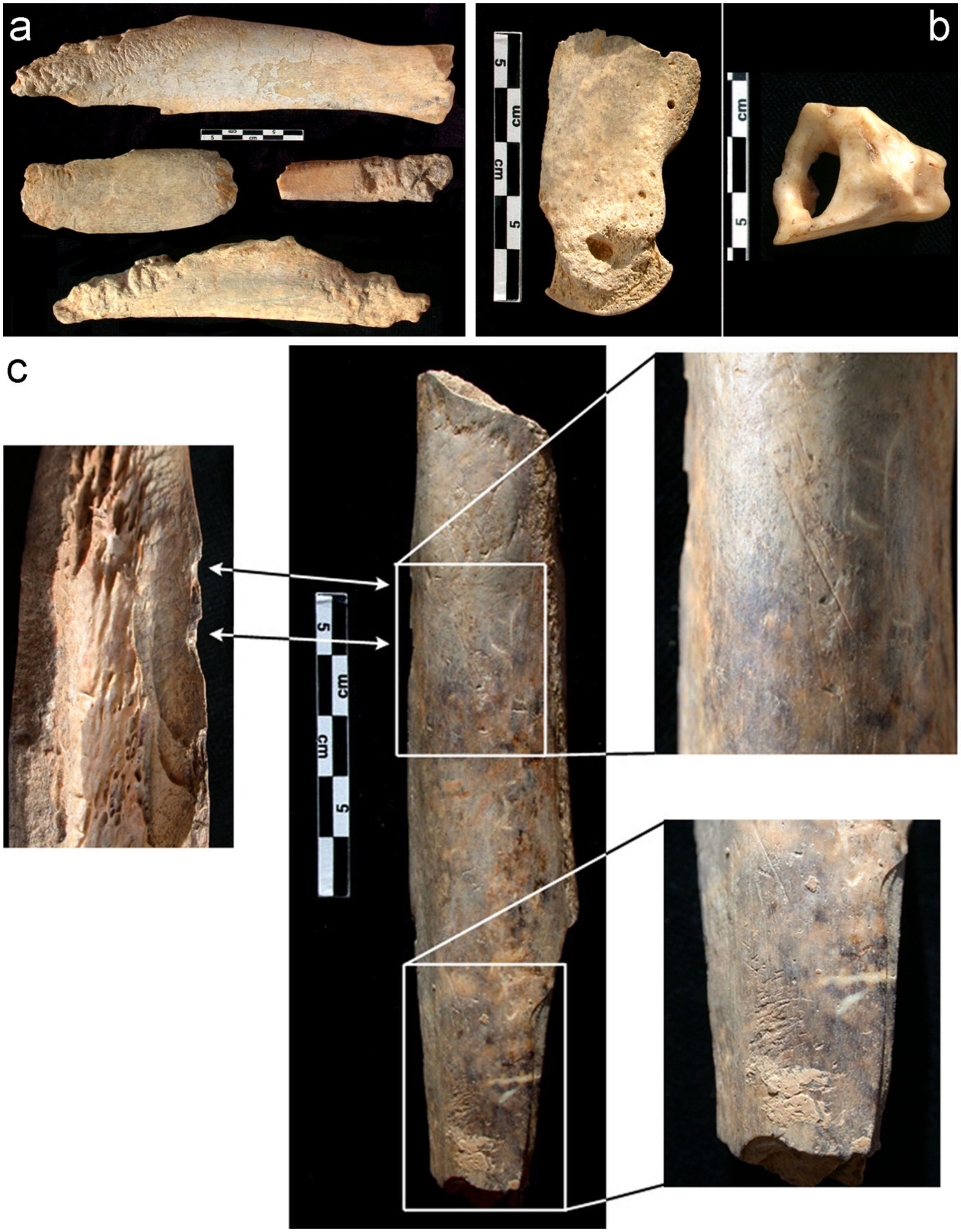

Supplementary Figure S5. Lithic and ivory tools found in Grotte des Gorges, US 2. The lithic assemblage comprises manufacturing by-products as well as debitage products. Blades and bladelets, mostly fragmented, were found including one with typical Aurignacian retouch. A massive flake retouched at its distal end is interpreted as an Aurignacian nose scraper. An elongated, flat fragment of ivory bearing polish and micro-chipping at one end was also found in US 2. Scale bar for the objects: 1 cm; scale bar for the middle close-up: 2 mm; scale bar for the close-up on the right: 5 mm.

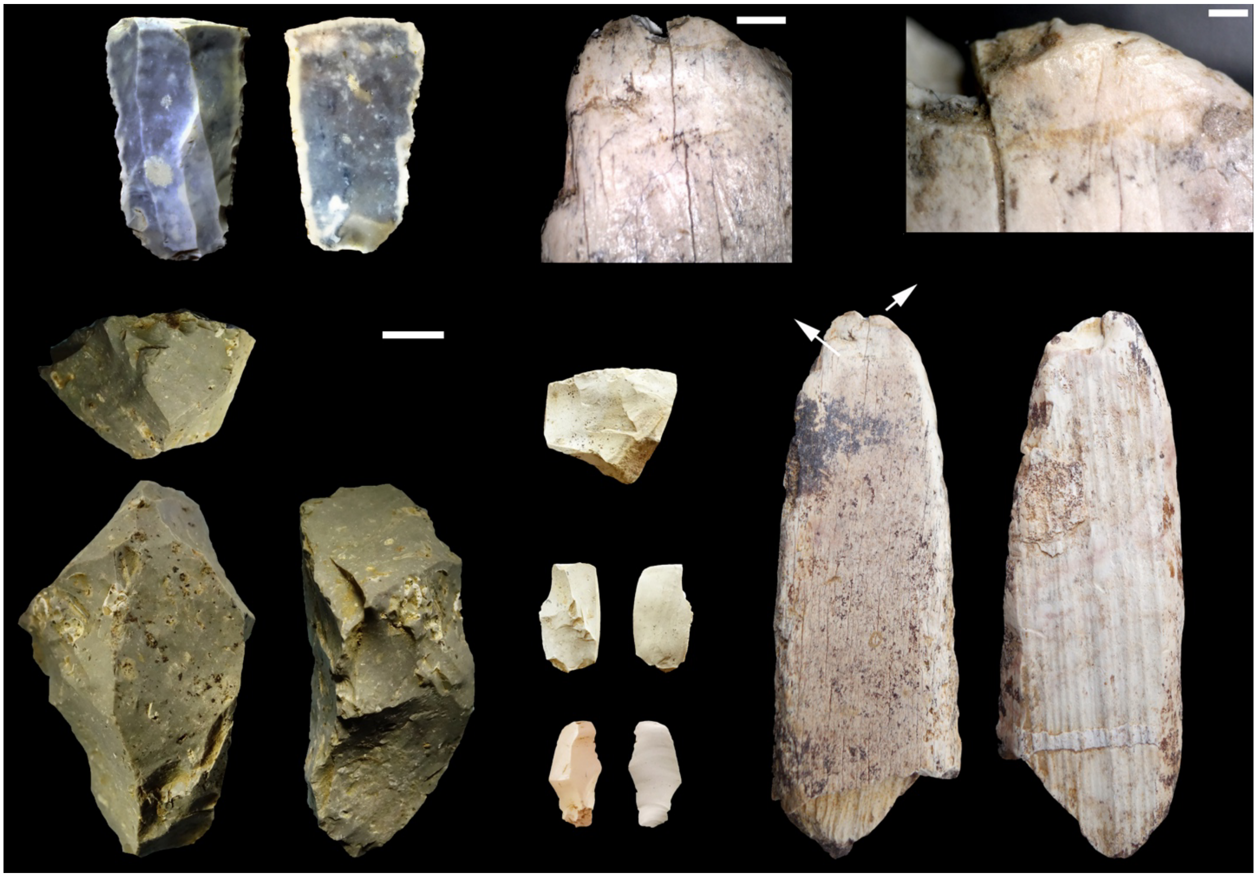

Supplementary Figure S6. Taxonomic and anatomical origin of the raw material used to make the Grotte des Gorges figurine. Reconstitution of an ammonite (a) and its comparison with a fossilized inner mould in lateral and ventral view (b) with the location of the phragmocone (*ph*), the body chamber (*bc*), the limit between the phragmocone and the body chamber (*), and the position of the septa (s1, s2, s3) each bounded by a suture line. The fragmentation of the phragmocone usually occurs along the suture lines bounding two septa (c). When observed in ventral view, the siphuncular tube position delimit an axis of bilateral symmetry that appears to have been exploited in the making of the Grotte des Gorges figurine (d). Unless indicated otherwise (a), scale bars = 1 cm.

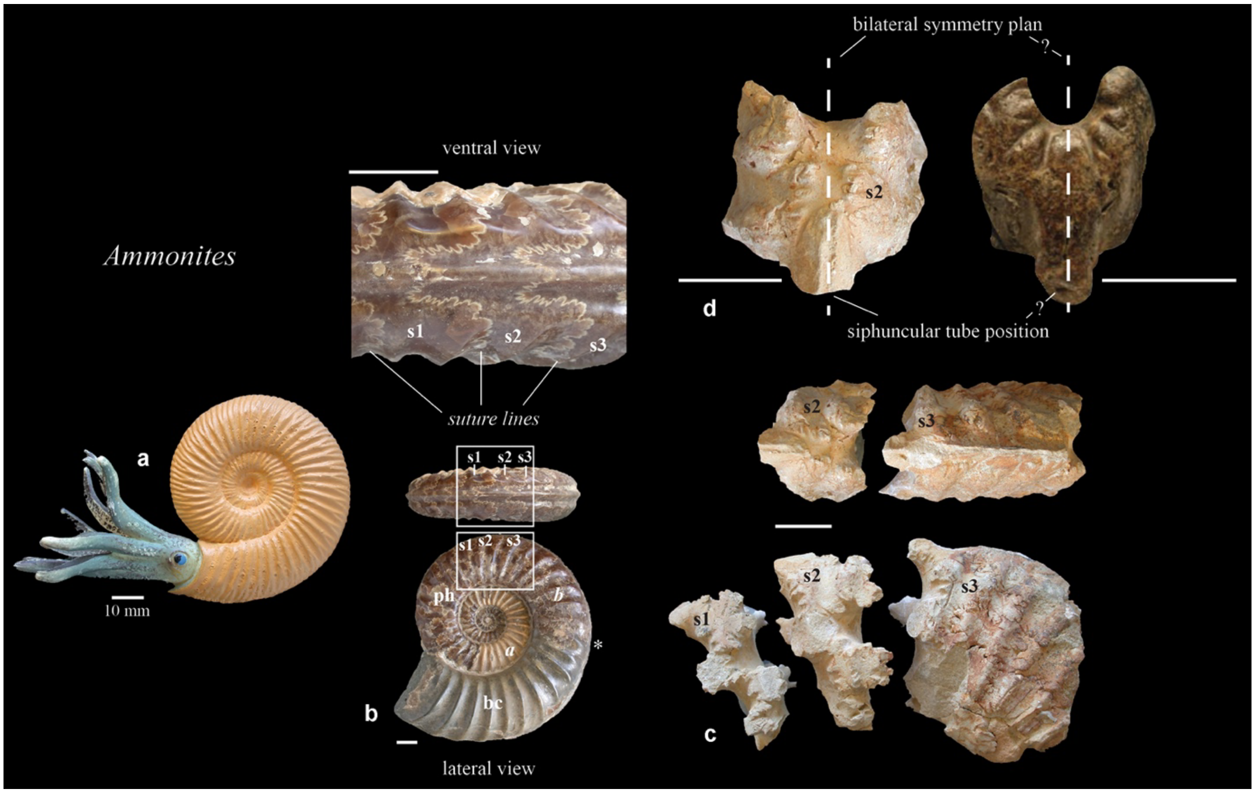

Supplementary Figure S7. Taxonomic identification of the ammonite. Based on the size and morphology of the object found at Grotte des Gorges, on its anatomical origin (a), and on documented ammonite species found in local limestone formations, the ammonite inner mould used to make the Grotte des Gorges figurine likely originates from a Pachyceratidae (b).

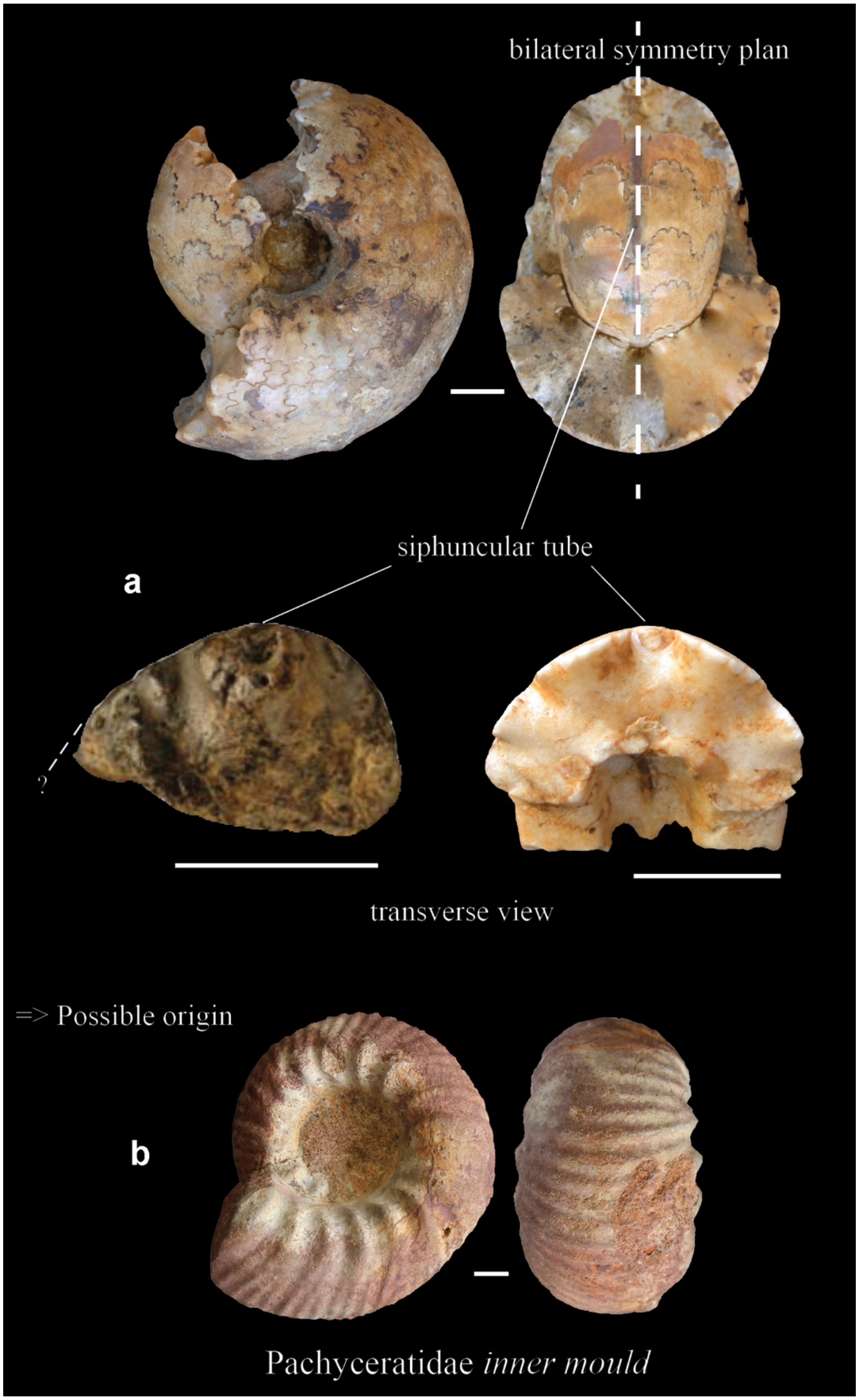

Supplementary Figure S8. Profiles of the notches arranged radially around the concave area on the Grotte des Gorges figurine. The locations of the profile acquisition are indicated on the top left 3D rendition of the concave area. Illustrations of the average profile for a 40-µm-thick band (a-g). Notches located on the left of the siphuncular tube position are skewed to the left (a-c) while those located on the right are skewed to the right (d-g). This pattern suggests the object was turned 180˚ between the production of each set.

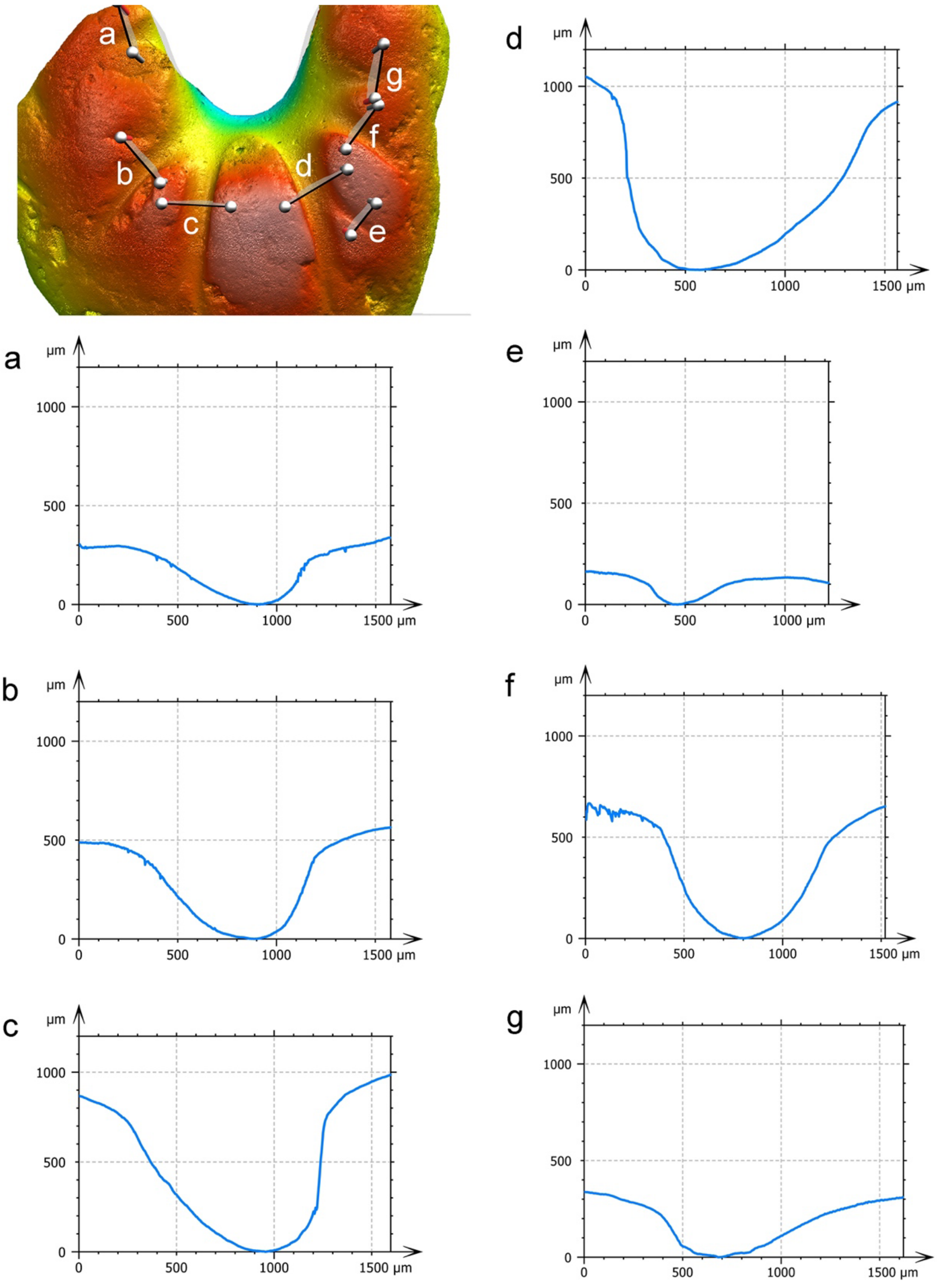

Supplementary Figure S9. Correlation matrix of textural parameters. Below the diagonal, scatterplots and trend lines for the values recorded for pairs of textural parameters. Above the diagonal, R^2 and significance, i.e., **: $0.001 > p \leq 0.01$, ***: $p \leq 0.001$, for each pair of textural parameters. See Supplementary Table S4 for details.

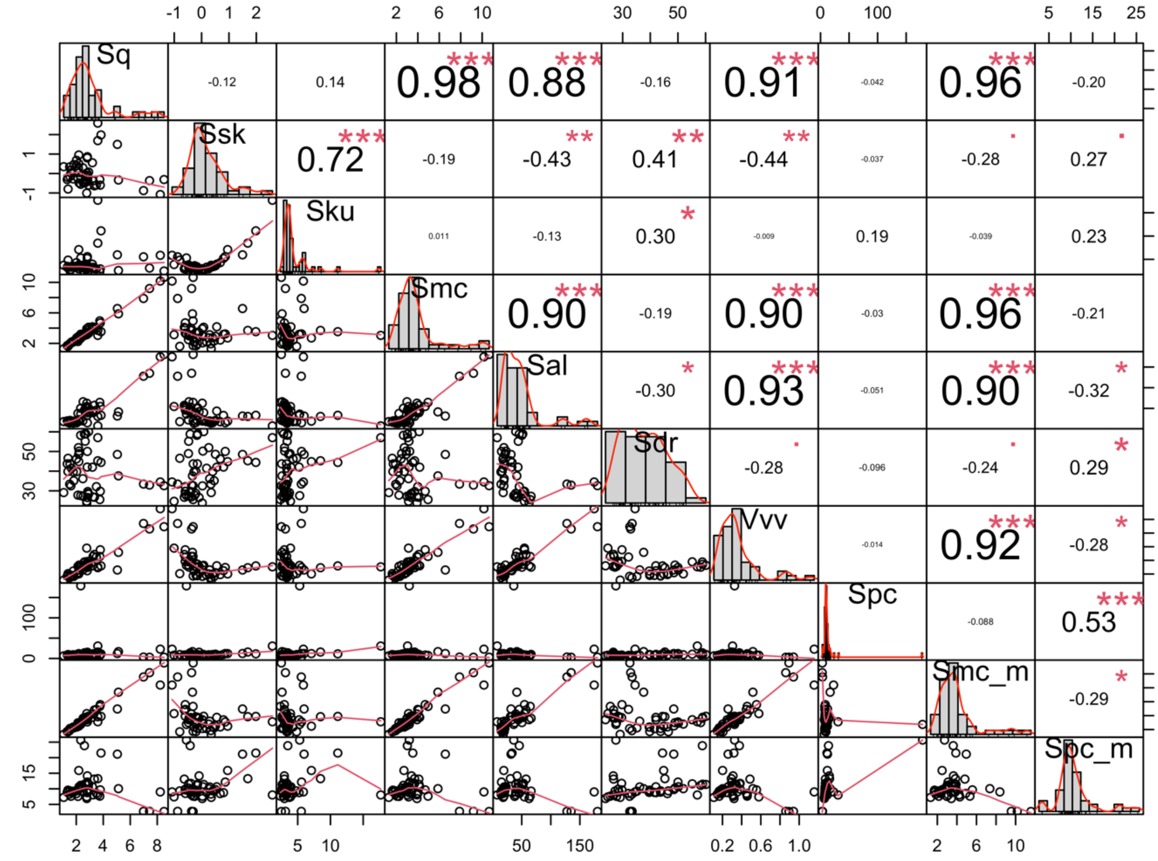

Supplementary Figure S10. Bayesian model produced using Chronomodel 1.5.0. Bayesian model (95% C.I., using IntCal20[27] calibration curve) of the Grotte des Gorges archaeological sequence (see SOM Table S6 for details).

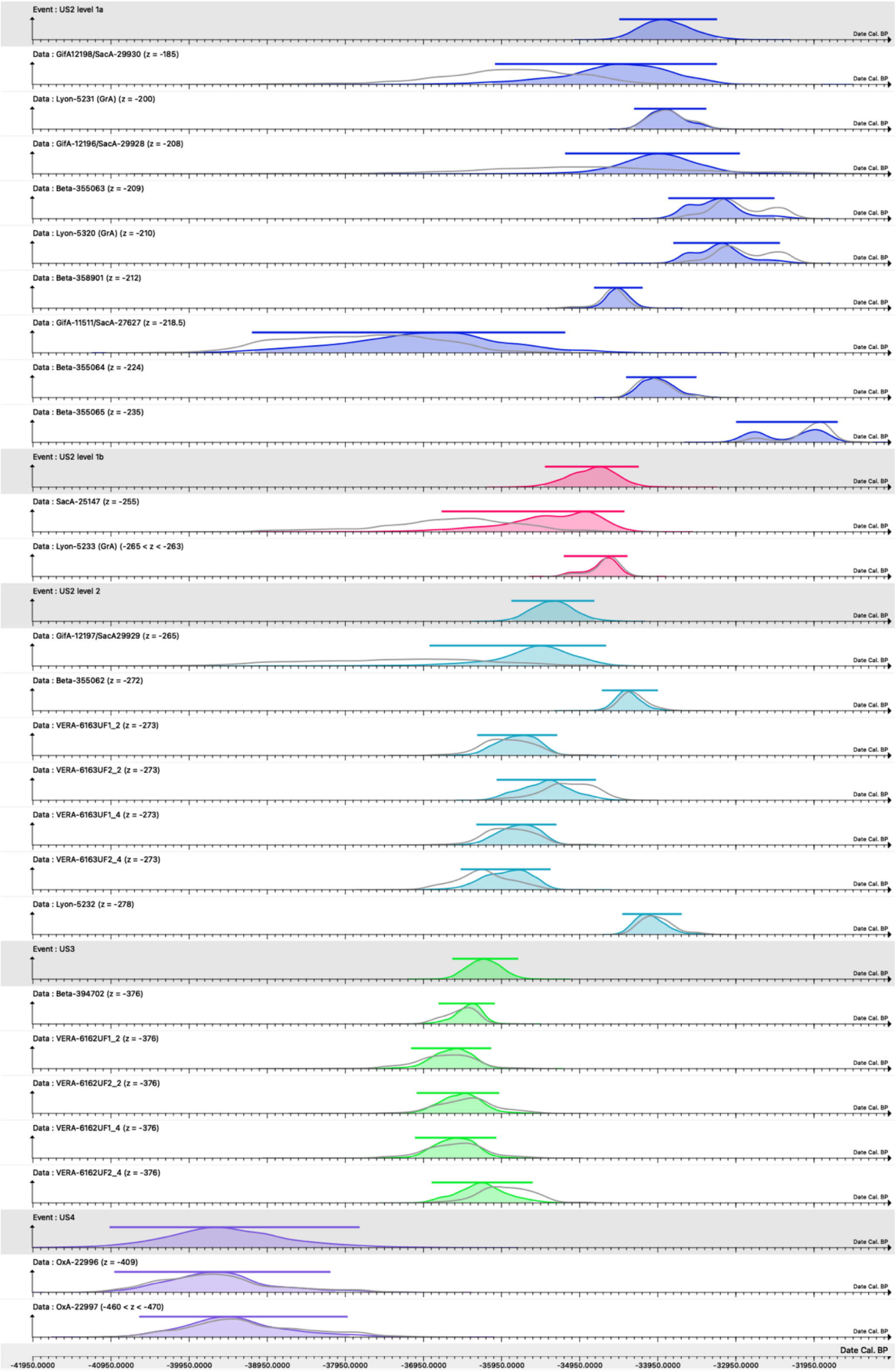

Supplementary Figure S11. Summary of the Bayesian model produced using Chronomodel 1.5.0. The model suggests calibrated age range (95% C.I., using IntCal20[27]) between 40,996 and 38,989 cal. BP for US 4, between 36,586 and 35,752 cal. BP for US 3, between 35,814 and 34,760 cal. BP for US 2 level 2, between 35,383 and 34,209 cal. BP for US 2 level 1b, and between 34,439 and 33,203 cal. BP for US 2 level 1a. (For details, see SOM Fig S10, SOM Table S6).

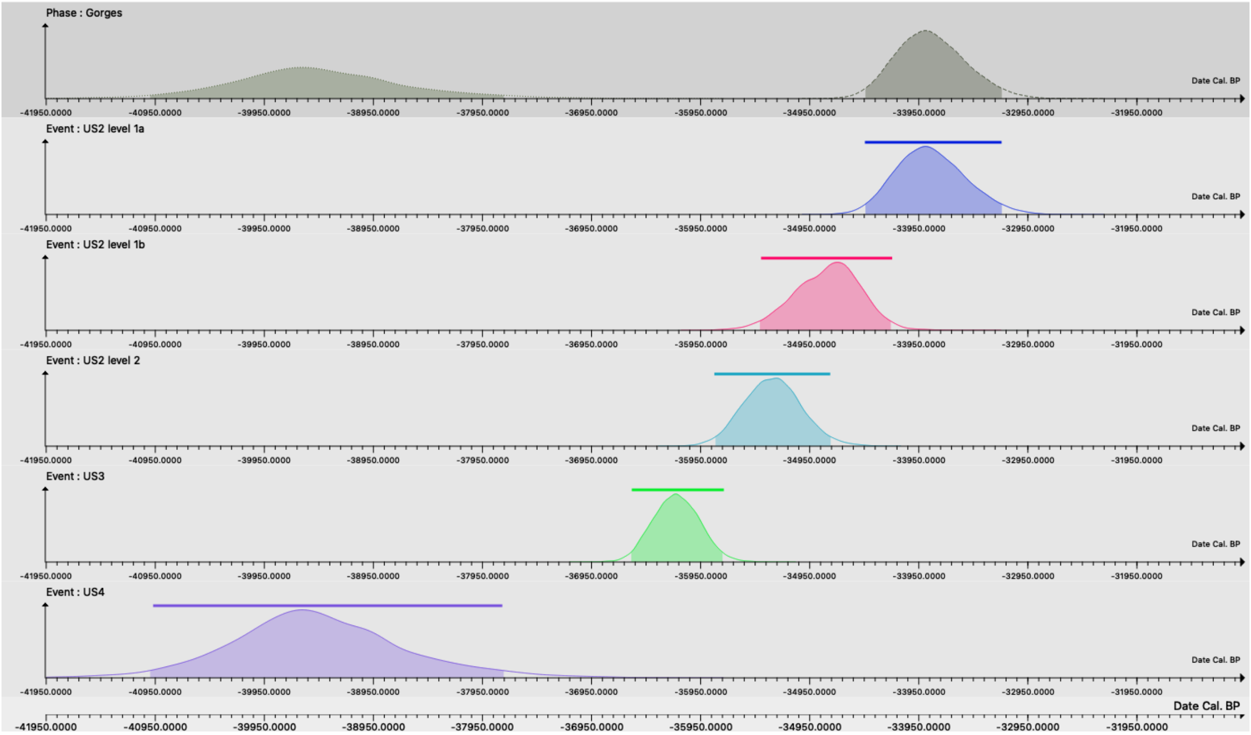

Supplementary Figure S12. Variations in the sedimentary sequence of the Grotte des Gorges. Matrix-supported diamicton characterising the lower sequence (a, b). Sandy lens interbedded in the middle part of the lower sequence (US 3) (c). Detail on the previous photo. Note the alternation of massive sandy units with bedded sandy-clay to silty-clay units (d, e). Current ripples in one of the interbedded sandy lenses within the lower sequence. Also note the pebble line to the left of the scale (f).

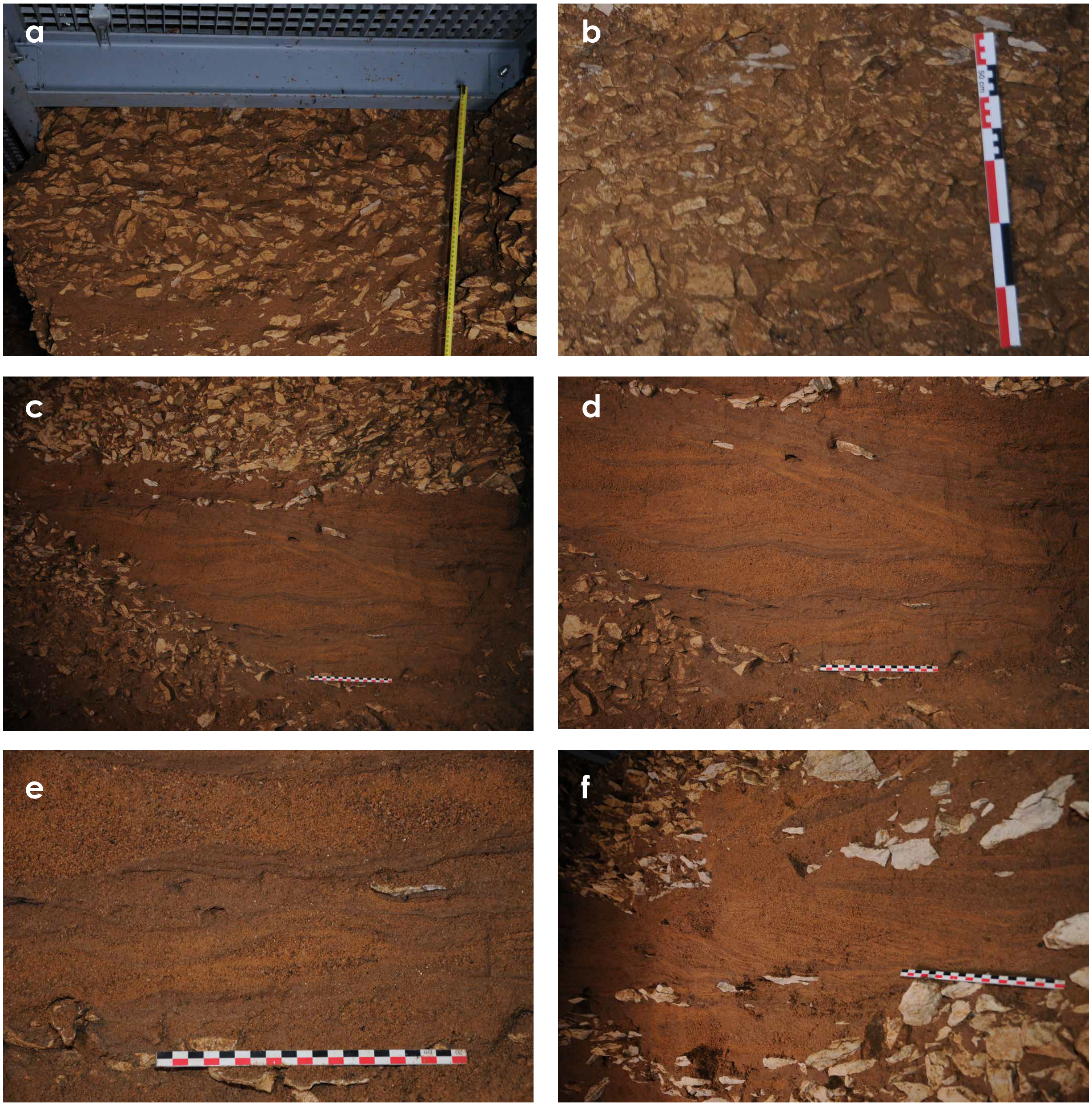

Supplementary Figure S13. Fabric on geligracts. (a) Woodcock[2] diagram with projections of the values of the lower sequence (natural clasts). (b) Fabrics of the natural clasts and projection of the values for the lower sequence (modified after Lenoble and Bertran[36]). (c) Rose diagram for orientation data from the lower sequence natural clasts (Benn IS index: 0.21; Benn IE index: 0.19; Rayleigh test: $n = 60$, $p = 0.3$, L = 13.91).

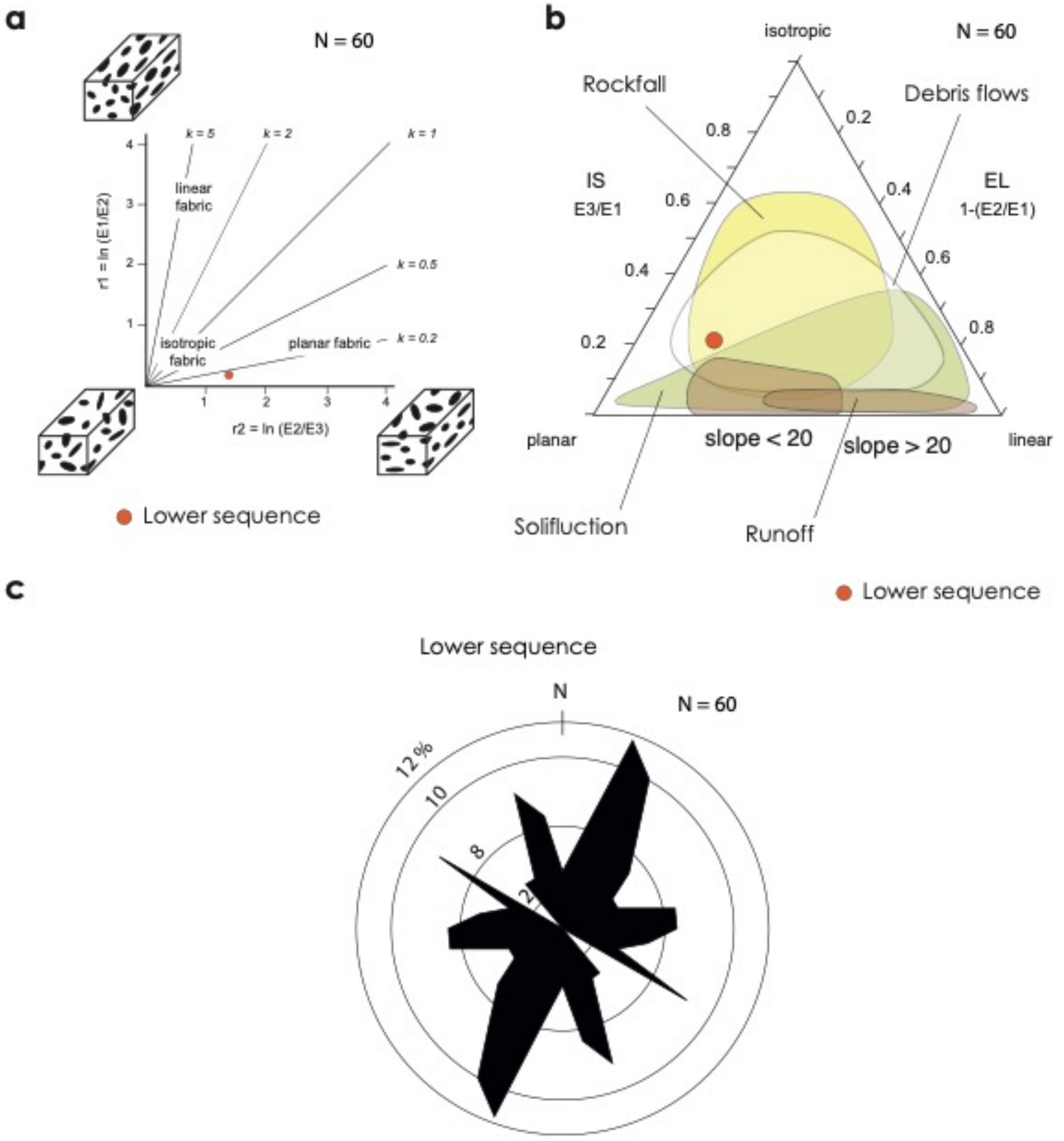

Supplementary Figure S14. Details of the Grotte des Gorges stratigraphy. Portion of section C17 to G 17. Above the limestone slab (LS), which rests on the top of the lower unit, a unit of yellow-brown silty clays and clayey silts and above brown to dark brown clays (a). Weathering horizon at the top of the lower sequence (b, c). Detail on the weathering horizon (d, e). Note the presence of an alteration cortex around some of the limestone pebbles or even a complete alteration of some (e).

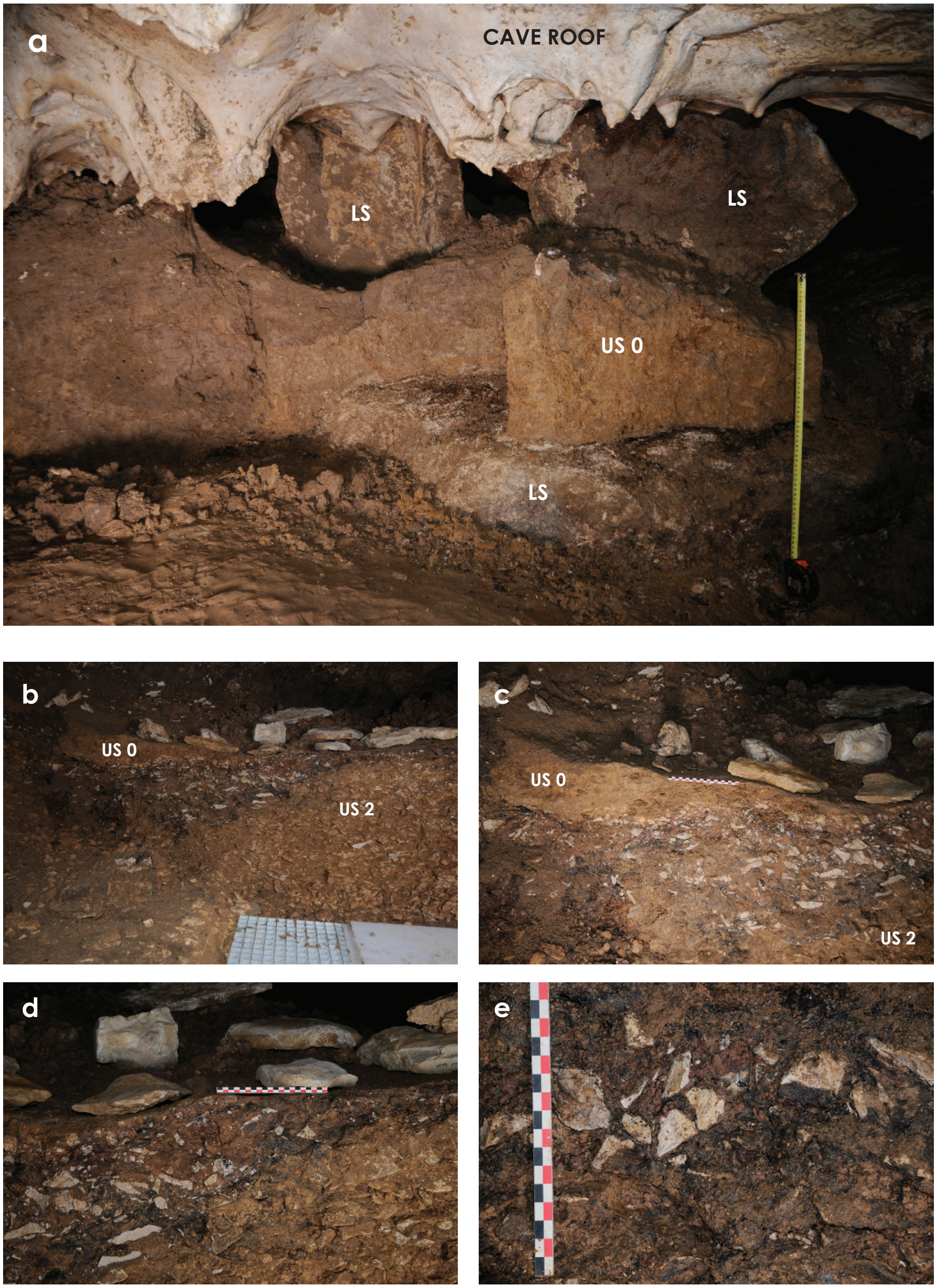

Supplementary Figure S15. Morphology of the terrain near the Grotte des Gorges entrance. Current small alluvial cone at the base of a small rocky escarpment on the eastern side of the Gorges valley. The sediments are transported through runoff on the slope and some of them penetrate karstic fissures located below the escarpment (1). Current small alluvial cone at the base of a diaclase allowing the evacuation of sediments from the plateau to the side of the Gorges valley. The photo was taken just upstream of the site, which actual entrance is indicated by the white arrow (2-3).

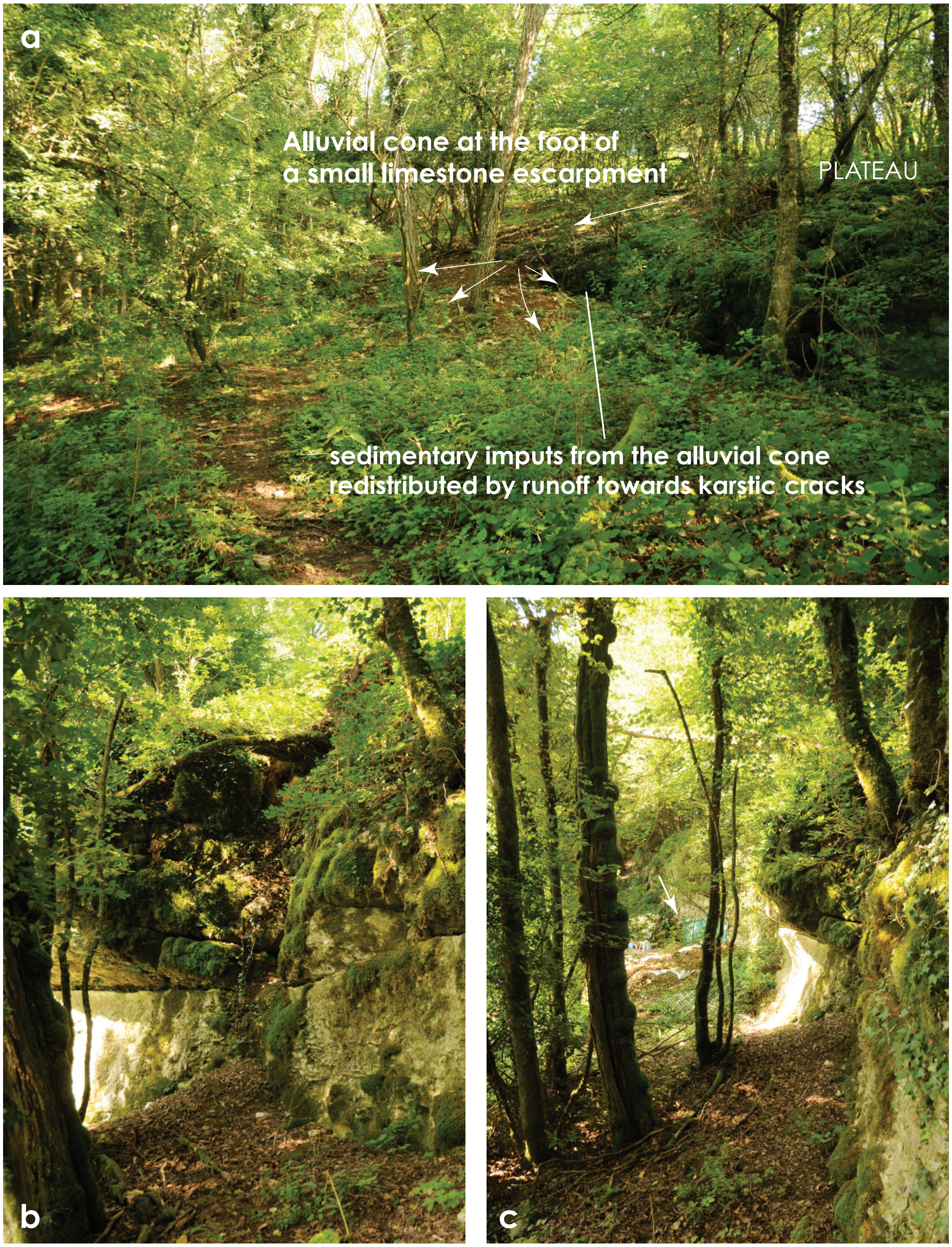

Supplementary Figure S16. Grotte des Gorges formation. Following an erosion phase of the colluvial spreading by the Gorges creek, a sinkhole in the limestone substratum facilitated debris flows, and runoff, which contributed to the formation of the cavity and the accumulation of the stratigraphic units.

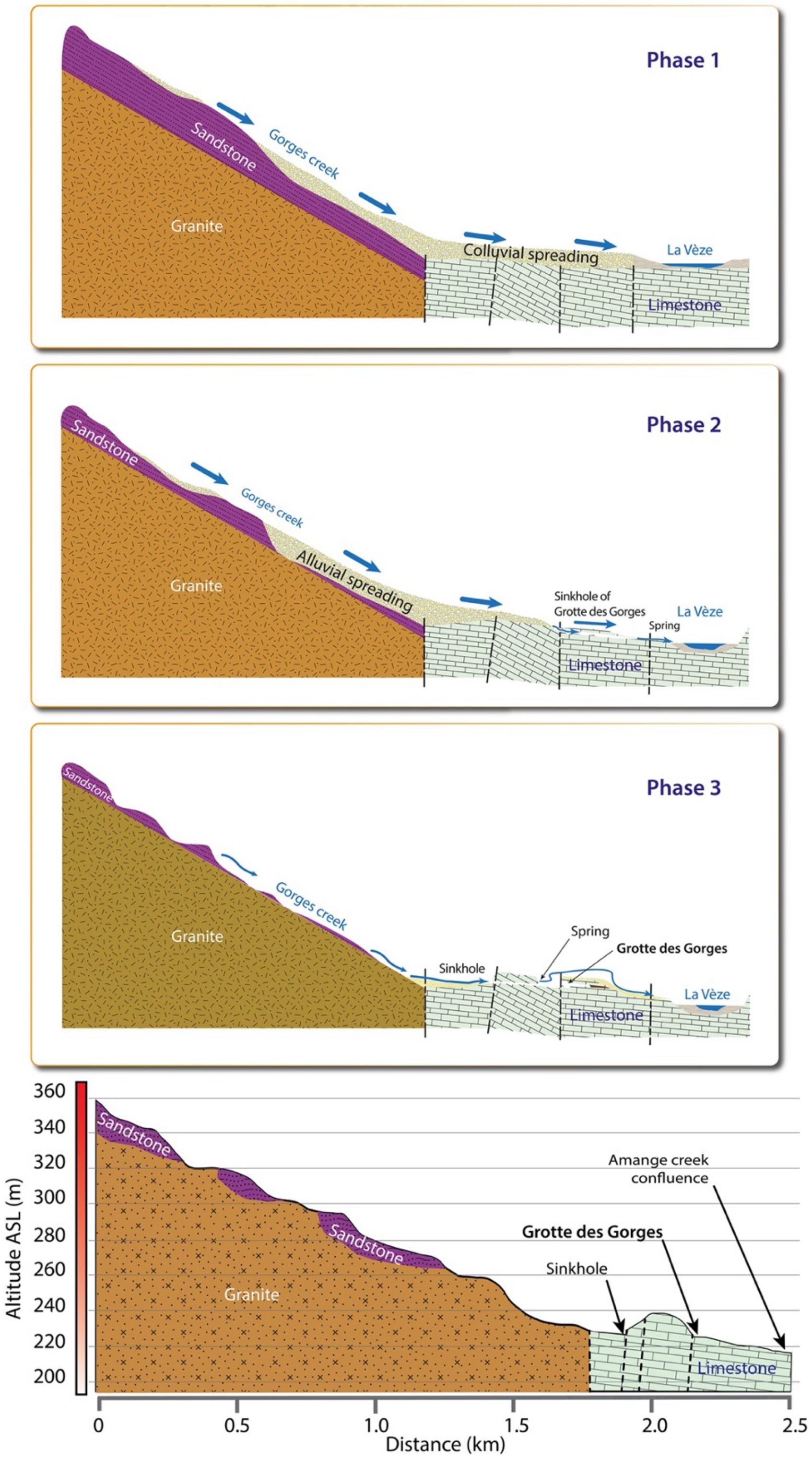

Supplementary Figure S17. Diffractogram showing the presence of abundant quartz and hydroxyapatite (calcium phosphate) in the crust surrounding altered limestone blocks. The low amount of calcite confirms that the carbonate has been altered and transformed into phosphate, probably by chemical dissolution/precipitation, or by epigeny, due to acid solutions derived from bat guano. The PDFXX-XXXX annotations are the reference numbers in the PDF-2 ICDD X-ray diffraction database. X-ray powder diffraction pattern was obtained using copper Kα (wavelength of 1.5406 Å).

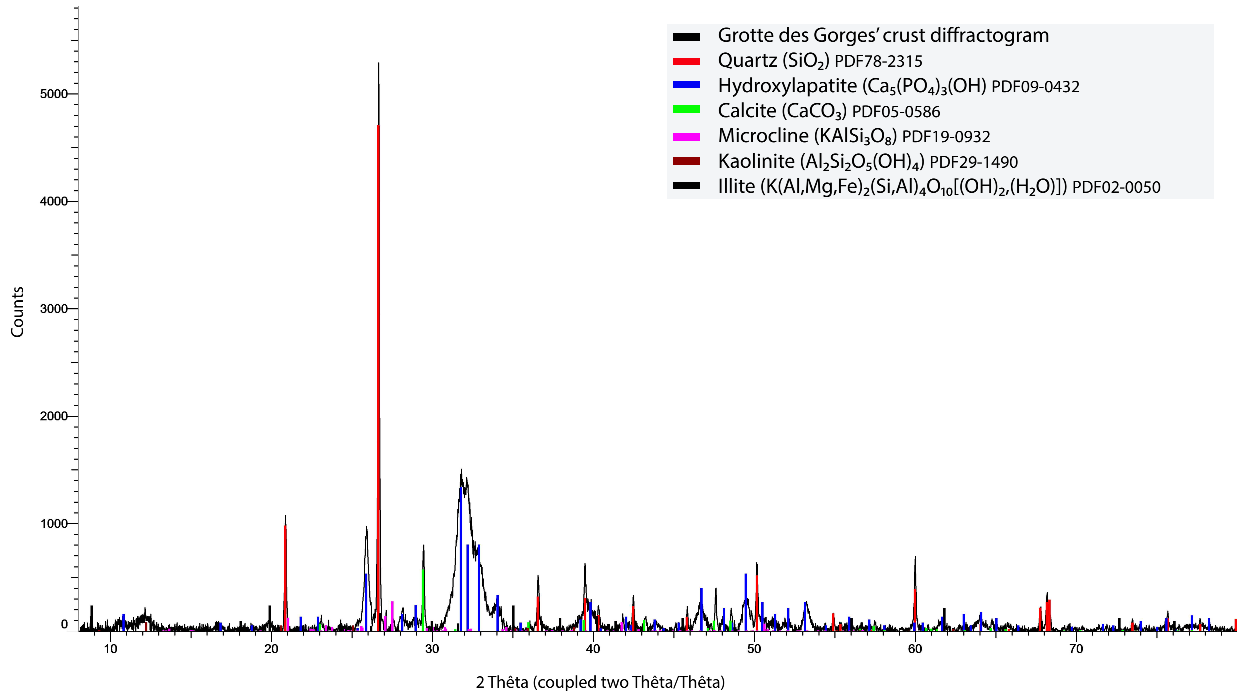

Supplementary Tables

Supplementary Table S1. Large mammal and avian species identified at Grotte des Gorges.

	**US 2**				**US 3**	**US 4**	**Total**
	Level 1a	Level 1b	Level 1b-2	Level 2			
Rodentia							
Marmota marmota				1			**1**
Lagomorpha							
Lepus sp.	3	1		1		3	**8**
Carnivora							
Ursus spelaeus	5			9		1	**15**
Meles meles				3			**3**
Vulpes vulpes	4	2					**6**
Canis lupus	2			1			
Crocuta spelaea	2	4	1			1	**8**
Felis sylvestris		1					**1**
Proboscidea							
Mammuthus primigenius	2					2	**4**
Perissodactyla							
Coelodonta antiquitatis	2						**2**
Equus ferus cf. *gallicus*	2	2	1	5	1		**11**
Artiodactyla							
Rangifer tarandus	90	7	1	57		5	**160**
Cervus elaphus	1			1		1	**3**
Capreolus capreolus		1					**1**
Indeterminate *Cervidae*	1			1			**2**
Bison priscus	9	60	11	284		3	**367**
Indeterminate *Ongulata*	5	7		30			**42**
Megafauna				10			**10**
Mesofauna				3			**3**
Aves							
Lagopus sp.	5			7			**12**
Lyrurus tetrix	1						**1**
Perdrix sp.	1						**1**
Columba sp.	2			1			**3**
**Total**	**137**	**85**	**14**	**414**	**1**	**16**	**667**

Supplementary Table S2. Number and percentage of identified skeletal elements by anatomical portion in US 2 from Grotte des Gorges. NISP: Number of Identified Specimens

	Level 1a		Level 1b		Level 2	
	NISP	%	NISP	%	NISP	%
**NISP**	**137**		**85**		**414**	
Isolated teeth	11	8.0	33	38.8	150	36.2
Cranial fragments	3	2.2	4	4.7	15	3.6
Mandible fragments	6	4.4	3	3.5	13	3.1
Vertebrae	3	2.2	1	1.2	13	3.1
Ribs	28	20.4	18	21.2	61	14.7
Long bone shafts	64	46.7	33	38.8	154	37.2
Long bone epiphyses	19	13.9	6	7.1	18	4.3
Carpal/Tarsal bones	4	2.9	1	1.2	4	1.0
Phalanges	2	1.5	1	1.2	5	1.2

Supplementary Table S3. Carnivore and anthropogenic modifications recorded on the large mammal bone assemblage from Grotte des Gorges.

	Poorly preserved surface	Carnivore modifications		Anthropogenic modifications			
		Gnawing	Digestion	Cut marks	Percussion notches	Retoucher	Burnt
US2 level	NR (%)	NR (%)	NR (%)	NR (%)	NR (%)	NR (%)	NR (%)
Level 1a	11 (8.59%)	5 (3.91%)	2 (1.56%)	4 (3.13%)	5 (3.91%)		2 (1.56%)
Level 1b	4 (4.71%)	18 (21.18%)	12 (14.12%)	2 (2.35%)	2 (2.35%)	1 (1.18%)	
Level 2	13 (3.20%)	46 (11.33%)	14 (3.45%)	3 (0.74%)	5 (1.23%)		17 (4.19%)

Note: Empty cells indicate absence of data.

Supplementary Table S4. Definition of the textural parameters (ISO 25178) used in the present study.

Parameter	Definition	Description	Unit
Sq	Root mean square height or quadratic mean height	Standard deviation of the height distribution relative to mean plane	µm
Ssk	Skewness	Skewness of the height distribution, or the degree of bias of the roughness shape (asperity).	no unit
Sku	Kurtosis	Kurtosis of the height distribution, or a measure of the sharpness of the roughness profile.	no unit
Sal	Auto-correlation length (s = 0.2)	Horizontal distance in the direction in which the auto-correlation function decays to the value of 0.2 the fastest.	µm
Sdr	Developed interfacial area ratio	Percentage of the definition area's additional surface area contributed by the texture as compared to the planar definition area. An *Sdr* of 0 indicates a completely level surface.	%
Smc	Inverse areal material ratio	Height above the mean plane at which the areal material ratio reaches 10%.	µm
Smc_m	Inverse areal material ratio (extracted on a z-mirror acquisition)	Depth below the mean plane at which the areal material ratio reaches 10%.	µm
Vvv	Dale void volume	Void volume of the valleys at 80% material ratio.	$µm^3/µm^2$
Spc	Arithmetic mean peak curvature	Arithmetic mean of the principal curvature of peaks on the surface. Small value indicates that the peaks have rounded shapes.	1/µm
Spc_m	Arithmetic mean valley curvature (extracted on a z-mirror acquisition)	Arithmetic mean of the principal curvature of valleys on the surface. Small value indicates that the valleys have rounded shapes.	1/µm

Supplementary Table S5. Textural data (ISO 25178). Values for the quadratic mean height *Sq*, the skewness *Ssk*, the kurtosis *Sku*, the auto-correlation length *Sal*, the developed interfacial area ratio *Sdr*, the inverse areal material ratio above the mean plane *Smc*, the dale void volume *Vvv*, the arithmetic mean peak curvature *Spc*, the inverse areal material ratio below the mean plane *Smc_m* and the arithmetic mean valley curvature *Spc_m*. *Smc_m* and *Spc_m* were calculated after mirroring the surface on its z- axis. See Supplementary Table S4 for definitions, descriptions, and units.

Acquisition_ID	State	Sq	Ssk	Sku	Smc	Sal	Sdr	Vvv	Spc	Smc_m	Spc_m
Gorges_acq01_2	A_NaturalSurface	7.42957386	-0.3647952	3.13033968	9.19040745	135.012166	32.9121455	0.86010612	2.99804163	10.1687529	2.76744034
Gorges_acq01_3	A_NaturalSurface	8.22550959	-1.0794236	5.86759914	10.1652078	174.674832	34.4896957	1.15684334	22.8490389	8.39228484	9.00100908
Gorges_acq01_4	A_NaturalSurface	6.98317799	-0.8761894	5.54753901	7.8874607	127.824301	33.424282	0.94053755	3.51677387	9.55709778	2.75724513
Gorges_acq01_5	A_NaturalSurface	8.50455431	-0.3091536	2.50497631	10.6511333	180.374027	32.6057582	0.8933391	3.17213199	11.5664482	2.72032537
Gorges_acq07_1	A_NaturalSurface	3.77961222	-0.5980457	2.95987297	4.68816762	54.7602693	29.7565695	0.5550457	8.34535596	5.583568	9.65979619
Gorges_acq07_2	A_NaturalSurface	2.12426463	-0.253186	3.64067589	2.24231265	37.8135457	27.7901938	0.31255229	8.33421516	2.97666912	7.55860402
Gorges_acq07_3	A_NaturalSurface	2.85217827	-0.3932938	3.34879759	3.70410683	52.8533153	29.6152844	0.38410843	8.34202197	3.72859136	11.7529242
Gorges_acq07_4	A_NaturalSurface	2.33299555	-0.5548397	5.30169596	3.20174027	36.2077806	27.5471778	0.32611178	178.761925	2.73811432	25.6587061
Gorges_acq07_5	A_NaturalSurface	3.3411537	-0.6319407	2.97210675	4.09552274	57.393084	26.7102484	0.48668642	8.47680928	4.8134097	13.1157311
Gorges_acq08_1	B_LustrousSurface	2.78035788	0.02379789	2.97808543	3.92144923	62.5660031	23.8014231	0.32041611	6.5615511	3.42243533	6.87926919
Gorges_acq08_2	B_LustrousSurface	3.24862453	-0.3738525	3.30292384	3.91899088	39.6690545	26.8765508	0.45871024	11.7086231	4.24032045	8.05310572
Gorges_acq08_3	B_LustrousSurface	2.77556994	-1.006583	3.89706326	3.13461696	54.5990833	24.6583672	0.57921806	7.95957729	4.29289753	7.41010898
Gorges_acq08_4	B_LustrousSurface	2.82241219	-0.0921836	2.95871603	3.40939213	41.9383395	26.6123247	0.31017888	15.3598517	3.65083361	14.1612856
Gorges_acq08_5	B_LustrousSurface	3.74460978	-0.3126235	2.36269762	4.95143406	64.5405489	25.5427328	0.45734023	7.39631294	5.32991735	7.63848047
Gorges_acq09_1	B_LustrousSurface	2.84842671	-0.211764	2.99268862	3.40712742	39.5013034	28.6785041	0.35414049	7.76329463	3.83660481	8.9643407
Gorges_acq09_2	B_LustrousSurface	5.12657855	-0.3339162	2.61336047	5.87511265	41.5421636	37.6000988	0.51735343	16.8318042	7.36695578	10.0523412
Gorges_acq09_3	B_LustrousSurface	2.91784953	-0.5984511	3.34450878	3.33624362	50.436211	28.3580264	0.38448542	7.70136756	3.91992614	9.86930912
Gorges_acq09_4	B_LustrousSurface	2.50856247	-0.4621275	2.63294249	2.98603951	66.0631339	27.2358893	0.30070735	9.92174513	3.62293173	9.26832649
Gorges_acq09_5	B_LustrousSurface	3.49157418	-0.5933926	2.94634076	3.99177342	45.811263	32.1256892	0.44912676	7.49832358	4.89717961	8.52424126
Gorges_acq11_1	C_BelowProtuberance	3.83366031	1.98276586	11.1213207	3.53287864	33.2650809	44.4357936	0.31902293	11.2862783	3.89134013	21.4913891
Gorges_acq11_2	C_BelowProtuberance	3.19635265	0.55370264	3.96204057	4.17301872	43.4748806	42.0109277	0.32054467	8.33261385	3.75328898	10.6499458
Gorges_acq11_3	C_BelowProtuberance	3.596483	1.68912746	8.48130674	3.69857857	30.5207194	45.2817597	0.29227324	15.7865092	3.85180947	13.2646712
Gorges_acq11_4	C_BelowProtuberance	2.81302731	0.80102703	5.2148536	3.18027293	32.8685234	42.0733696	0.24770908	10.051387	3.40205233	8.7660948
Gorges_acq11_5	C_BelowProtuberance	5.04596139	1.49694281	5.97355913	6.56565079	31.5562978	48.4953646	0.2587422	12.1728732	4.95681011	21.1357867
Gorges_acq10_1	D_Concavity	2.66075522	-0.4725546	3.38587599	3.17564429	44.0383653	55.187882	0.37847619	10.4410738	3.41283713	11.6031043
Gorges_acq10_2	D_Concavity	2.91368924	0.07602814	2.63028136	4.11420565	39.6816872	60.1072641	0.32728258	9.7554362	3.910519	11.4096879
Gorges_acq10_3	D_Concavity	2.60348022	-0.4179805	3.48988358	3.02494605	45.7516292	58.846858	0.37611855	13.9079849	3.62559905	23.8581715
Gorges_acq10_4	D_Concavity	2.32730929	-0.1698805	3.41781173	2.65756053	50.3013353	58.238523	0.29978494	9.41707939	3.00228109	11.1181369
Gorges_acq10_5	D_Concavity	2.70709017	-0.1471916	2.85884371	3.46425692	51.7352953	58.9431385	0.3421153	9.71774314	3.76181641	11.487339
Gorges_acq29_1	E_Notches	1.38285299	-0.2043622	3.48077862	1.67611464	16.2448398	39.7980524	0.17955911	8.35403322	1.71485203	9.57102254
Gorges_acq29_2	E_Notches	2.82184935	0.865379	5.58819208	3.24059365	15.1526004	50.0250174	0.29549323	12.1401592	3.0970015	12.1665263
Gorges_acq29_3	E_Notches	1.33062044	-0.3080926	3.2957271	1.70754085	19.884024	43.7162412	0.17040302	8.65395947	1.73630812	8.92515206
Gorges_acq29_4	E_Notches	3.59649847	2.58835505	17.682929	3.04487729	7.67411394	57.0259012	0.30534332	30.6593211	3.1195049	8.02564207
Gorges_acq29_5	E_Notches	1.53382816	-0.1548172	2.55924952	1.91958168	20.9173715	43.738622	0.17480432	8.54008766	2.15968803	9.35070457
Gorges_acq30_1	E_Notches	2.18690142	0.95205786	7.09124973	2.70639186	19.0772342	44.8414843	0.20127175	11.9549923	2.48737148	15.8869378
Gorges_acq30_2	E_Notches	2.03494184	0.40384382	3.42483985	2.73303243	15.1868212	39.7845061	0.19838499	9.65369586	2.36145792	9.35297406
Gorges_acq30_3	E_Notches	2.0120237	0.68073938	4.29821555	2.43387197	17.674224	46.2128535	0.19137134	7.75884501	2.37944954	8.46024584
Gorges_acq30_4	E_Notches	1.77630304	-0.2791394	3.37213785	2.16947901	14.1691266	39.6083346	0.24386901	9.18633879	2.36020725	8.86017883
Gorges_acq30_5	E_Notches	1.64553013	0.18343002	3.31587401	2.1704885	17.774108	41.4791537	0.17988435	8.04736988	2.1267756	8.83861088
Gorges_acq31_1	E_Notches	1.39281445	-0.7976647	4.83469655	1.61184883	17.9771981	46.5464644	0.20558812	9.58272493	1.68567769	9.33634238
Gorges_acq31_2	E_Notches	2.18229997	0.50696623	3.62408736	2.92898787	20.9339823	48.5605925	0.21907901	10.5833845	2.52513999	9.76401463
Gorges_acq31_3	E_Notches	2.21211809	-0.4803048	3.82153925	2.48951766	21.8933228	48.9348306	0.33049373	10.6352416	2.9886108	10.2445438
Gorges_acq31_4	E_Notches	2.16223715	0.26228544	2.98295884	2.73432653	19.2525582	51.958506	0.20183774	9.10885444	2.78570643	10.0489312
Gorges_acq31_5	E_Notches	2.20897369	0.04765738	2.99932208	2.9172065	22.9554352	50.1871791	0.25854889	10.1744802	2.92781897	10.6670148
Gorges_acq32_1	E_Notches	1.50824314	0.33901488	3.33134711	1.93975886	14.345986	33.3568836	0.14579384	7.29043511	1.85108538	8.29271291
Gorges_acq32_2	E_Notches	2.4882629	0.29567784	3.26630269	3.13194012	19.7217132	36.1664687	0.2601815	7.85999731	3.20284724	10.6064531
Gorges_acq32_3	E_Notches	1.06975451	0.35151728	3.73510443	1.29188548	18.0732003	29.0998493	0.11040474	6.35521034	1.31273344	7.23568879
Gorges_acq32_4	E_Notches	1.60140874	0.03121652	2.82106933	2.06114697	19.2544104	32.5234158	0.16583204	7.27501421	2.14899449	7.60548459
Gorges_acq32_5	E_Notches	1.81526525	0.45920241	3.50475312	2.31957045	19.2070226	35.1495139	0.16483169	7.77921218	2.24200952	9.23333889

Supplementary Table S6: AMS radiocarbon dating results (outliers in red).

Lab. ID	Sample	US, level	M^2 (depth)	^{14}C-age (BP)	Calibrated age BP (95% C.I.)
Beta-319487	Human femur	US2, top	N6 (z = -200)	5620 ± 30	6482–6469 (2.9%) 6452–6310 (91.9%)
GifA 12198/ SacA-29930	Bison radio-ulna	US 2, 1a	J10 (z = -185)	31390 ± 880	38051–37924 (0.5%) 37843–34072 (94.5%)
Lyon-5231 (GrA)	Long bone fragment	US 2, 1a	J10 (z = -200)	29240 ± 170	34233–33306
GifA-12196/ SacA-29928	Reindeer metatarsal	US 2, 1a	I10 (z = -208)	30500 ± 1300	37673–31671
Beta-355063	Tibia fragment (large mammal)	US 2, 1a	M6 (z = -209)	28650 ± 160	33561–32174
Lyon-5230 (GrA)	Long bone fragment	US 2, 1a	J10 (z = -210)	28620 ± 160	33489–32130
Beta-358901	Reindeer antler	US 2, 1a	M8 (z = -212)	30100 ± 180	35013–34979 (0.7%) 34920–34191 (94.3%)
GifA-11511/ SacA-27627	Hyena mandible	US 2, 1a	F13 (z = -218.5)	33030 ± 750	39552–36080
Beta-355064	Reindeer humerus	US 2, 1a	M6 (z = -224)	29430 ± 190	34400–33511
Beta-355065	Reindeer tibia	US 2, 1a	N6 (z = -235)	28010 ± 170	32854–32431 (12.8%) 32328– 31546 (82.2%)
SacA-25148	Bison humerus	US 2, 1b	G13 (z = -248)	19510 ± 170	23813–23072
SacA-25147	Reindeer femur	US 2, 1b	F12 (z = -255)	32100 ± 710	38757–35079
Lyon-5233 (GrA)	Long bone fragments (N = 3)	US 2, 1b	J10 (-265 ≤ z ≥ -263)	30190 ± 180	35107–34292
GifA-12197/ SacA-29929	Reindeer metapodial	US 2, 2	E12 (z = -265)	32600 ± 1000	39648–35151
Beta-355062	Bison radius	US 2, 2	I13 (z = -272)	29740 ± 200	34587–33882
VERA-6163UF1_2[1]*	Long bone fragment	US 2, 2	I10 (z = -273)	31570 +350/-340	36553–35223
VERA-6163UF2_2[1]*	Long bone fragment	US 2, 2	I10 (z = -273)	30730 +350/-330	35824–34461
VERA-6163UF1_4[1]*	Long bone fragment	US 2, 2	I10 (z = -273)	31510 +340/-320	36471–35228
VERA-6163UF2_4[1]*	Long bone fragment	US 2, 2	I10 (z = -273)	31850 +340/-320	36877–35473
Lyon-5232 (GrA)	Bone	US 2, 2	I11 (z = -278)	29390 ± 170	34357–33507
Beta-394702[2]	Horse rib	US 3	F12 (z = -376)	32120 ± 190	36894–36117
VERA-6162UF1_2[2]*	Horse rib	US 3	F12 (z = -376)	32300 +360/-350	37532–35893
VERA-6162UF2_2[2]*	Horse rib	US 3	F12 (z = -376)	32030 +360/-340	37091–35576
VERA-6162UF1_4[2]*	Horse rib	US 3	F12 (z = -376)	32170 +380/-360	37387–35714
VERA-6162UF2_4[2]*	Horse rib	US 3	F12 (z = -376)	31570 +360/-350	36620–35231
OxA-22996	Long bone fragment	US 4	G10 (z = -409)	34550 ± 600	40932–37948
OxA-22997	Long bone fragment	US 4	G10 (-470 ≤ z ≥ -460)	34250 ± 550	40540–37609

* Ultrafiltration method.

[1] Same sample dated multiple times.

[2] Same sample dated multiple times.

Supplementary References

1. Campy, M., Chauve, P. & Pernin, C. Pesmes, Carte géologique de la France (1/50 000) n° 501 et Notice explicative. (1983).
2. Woodcock, N. H. Specification of fabric shapes using an eigenvalue method. *Geol. Soc. Am. Bull.* **88**, 1231–1236 (1977).
3. Curray, J. R. The analysis of two-dimensional orientation data. *J. Geol.* **64**, 117–131 (1956).
4. Bertran, P. *et al.* Dépôts de pente continentaux: Dynamiques et faciès. *Quaternaire* **Hors Série**, 1–259 (2004).
5. Bertran, P. & Texier, J.-P. Structures sédimentaires d'un cǒne de flots de débris (Vars, Alpes françaises méridionales). *Permafr. Periglac. Process.* **5**, 155–170 (1994).
6. van Steijn, H., Bertran, P., Francou, B., Texier, J.-P. & Hétu, B. Models for the genetic and environmental interpretation of stratified slope deposits: Review. *Permafr. Periglac. Process.* **6**, 125–146 (1995).
7. Moss, A. J. & Walker, P. H. Particle transport by continental water flows in relation to erosion, deposition, soils, and human activities. *Sediment. Geol.* **20**, 81–139 (1978).
8. Ferrier, C. Le contexte environnemental du peuplement paléolithique de Bulgarie du Nord : Le karst de Karlukovo et ses dépôts. (PhD Thesis, Université de Bordeaux 1, 1994).
9. Karkanas, P., Rigaud, J.-P., Simek, J. F., Albert, R. M. & Weiner, S. Ash bones and guano: A study of the minerals and phytoliths in the sediments of Grotte XVI, Dordogne, France. *J. Archaeol. Sci.* **29**, 721–732 (2002).
10. Queffelec, A., Bertran, P., Bos, T. & Lemée, L. Mineralogical and organic study of bat and chough guano: Implications for guano identification in ancient context. *J. Cave Karst Stud.* **80**, 49 (2018).
11. Guadelli, J.-L. Étude taphonomique du repaire d'hyènes de Camiac (Gironde, France). Éléments de comparaison entre un site naturel et un gisement préhistorique. *Bull. Assoc. Fr. Pour Létude Quat.* **26**, 91–100 (1989).
12. Fosse, P. Le rôle de l'hyène dans la formation des associations osseuses : 150 ans de controverses. L'apport des anciens textes de préhistoire et de paléontologie du Quaternaire aux études taphonomiques actuelles. *Paléo Rev. Archéologie Préhistorique* **7**, 49–84 (1995).
13. Fosse, P. La grotte n° 1 de Lunel-Viel (Hérault, France) : Repaire d'hyènes du Pléistocène moyen. Étude taphonomique du matériel osseux. *Paléo Rev. Archéologie Préhistorique* **8**, 47–79 (1996).
14. Fosse, P. Variabilité des assemblages osseux créés par l'Hyène des Cavernes. *Paléo Rev. Archéologie Préhistorique* **9**, 15–54 (1997).
15. Delagnes, A. *et al.* Le gisement Pléistocène moyen et supérieur d'Artenac (Saint-Mary, Charente) : Premier bilan interdisciplinaire. *Bull. Société Préhistorique Fr.* **96**, 469–496 (1999).
16. Monchot, H. Un assemblage original au Paléolithique Moyen : Le repaire á Hyènes, porcs-épics et hominidés de la grotte Geula (Mont Carmel, Israël). *Paléorient* **31**, 27–42 (2005).
17. Baca, M. *et al.* Ancient DNA of narrow-headed vole reveal common features of the Late Pleistocene population dynamics in cold-adapted small mammals. *Proc. R. Soc. B Biol. Sci.* **290**, 20222238 (2023).
18. Hernández Fernández, M. & Peláez-Campomanes, P. The bioclimatic model: A method of palaeoclimatic qualitative inference based on mammal associations. *Glob. Ecol. Biogeogr.* **12**, 507–517 (2003).

19. Royer, A., García Yelo, B. A., Laffont, R. & Hernández Fernández, M. New bioclimatic models for the quaternary palaearctic based on insectivore and rodent communities. *Palaeogeogr. Palaeoclimatol. Palaeoecol.* **560**, 110040 (2020).
20. Arbez, L. *et al.* The missing Myopus. Plugging the gaps in Late Pleistocene small mammal identification in western Europe with geometric morphometrics. *J. Quat. Sci.* **36**, 224–238 (2021).
21. Affolter, J. Provenance des silex préhistoriques du Jura et des régions limitrophes. (PhD Thesis, Université de Neuchâtel and École Pratique des Hautes-Études de Paris, 2022).
22. Fischer, L. E., Harris, S. K., Affolter, J. & Knipper, C. Linking quarry and settlement on the Swabian Alb, Southern Germany. *Quarry E-Newsl. SAAs Prehist. Quarr. Early Mines Interest Group* **10**, 8–20 (2013).
23. Bressy-Leandri, C. Caractérisation et gestion du silex des sites mésolithiques et néolithiques du Nord-Ouest de l'arc alpin. Une approche pétrographique et géochimique. (PhD Thesis, Université de Provence - Aix-Marseille I, 2002).
24. Brandl, M. The multi layered chert sourcing approach (mla) analytical provenance studies of silicite raw materials. *Archeometriai Műh.* **13**, 145–156 (2016).
25. Fernandes, P. Itinéraires et transformations du silex : Une pétroarchéologie refondée, application au Paléolithique moyen. (PhD Thesis, Université de Bordeaux 1, 2012).
26. Pigeau, R. L'art parietal. in *La Grotte des Gorges, Amange (Jura) : Bilan de sondages et de fouille programmée 2008-2014* (ed. David, S.) 195–245 (Service Régional d'Archéologie Franche-Comté, 2014).
27. Pigeau, R. L'art mobilier : les blocs gragés. in *La Grotte des Gorges, Amange (Jura) : Bilan de sondages et de fouille programmée 2008-2014* (ed. David, S.) 246–337 (Service Régional d'Archéologie Franche-Comté, 2014).
28. Vanhaeren, M. & d'Errico, F. Aurignacian ethno-linguistic geography of Europe revealed by personal ornaments. *J. Archaeol. Sci.* **33**, 1105–1128 (2006).
29. Mouton, P. & Joffroy, R. *Le gisement aurignacien des Rois à Mouthiers (Charente)*. (CNRS, 1958).
30. Dutkiewicz, E. *Zeichen. Markierungen, Muster und Symbole im Schwäbischen Aurignacien*. (Kerns Verlag, 2021).
31. Man-Estier, E. Les Ursidés au naturel et au figure pendant la Préhistoire. (PhD Thesis, Muséum national d'histoire naturelle - Paris, 2009).
32. Gárate Maidagán, D. & González Sainz, C. Micolón. in *Las cuevas con arte paleolítico en Cantabria* (eds. Malpelo García, B. & Castanedo Tapia, I.) 77–83 (Asociación Cántabra para la Defensa del Patrimonio Subterráneo, 2010).
33. González-Sainz, C., Ruiz-Redondo, A., Garate-Maidagan, D. & Iriarte-Avilés, E. Not only Chauvet: Dating Aurignacian rock art in Altxerri B Cave (northern Spain). *J. Hum. Evol.* **65**, 457–464 (2013).
34. Clottes, J. *La grotte Chauvet. L'art des origines*. (Le Seuil, 2001).
35. Baffier, D. & Girard, M. La Grande Grotte d'Arcy-sur-Cure (Yonne), nouveau sanctuaire paléolithique. Résultats préliminaires. *Rev. Archéologique Est Cent.-Est* **43**, 195–205 (1992).
36. Lenoble, A. & Bertran, P. Fabric of Palaeolithic levels: Methods and implications for site formation processes. *J. Archaeol. Sci.* **31**, 457–469 (2004).
